# Machine learning in the prediction of treatment response for emotional disorders: A systematic review and meta-analysis

**DOI:** 10.1016/j.cpr.2025.102593

**Published:** 2025-05-22

**Authors:** Joshua Curtiss, Christopher DiPietro

**Affiliations:** a Center for Cognitive and Brain Health, Department of Applied Psychology, Northeastern University, United States of America; b Psychiatry Department, Massachusetts General Hospital/Harvard Medical School, United States of America

**Keywords:** Precision medicine, Machine learning, Treatment, Anxiety, Depression, Meta-analysis

## Abstract

**Background:**

Emotional disorders such as depression and anxiety affect millions globally and pose a significant burden on public health. Personalized treatment approaches using machine learning (ML) to predict treatment response could revolutionize treatment strategies. However, there is limited evidence as to whether ML is successful in predicting treatment outcomes. This meta-analysis aims to evaluate the accuracy of ML algorithms in predicting binary treatment response (responder vs. non-responder) to evidence-based psychotherapies, pharmacotherapies, and other treatments for emotional disorders, and to examine moderators of prediction accuracy.

**Methods:**

Following PRISMA guidelines, a comprehensive literature search was conducted across PubMed and PsycINFO from January 1st, 2010 to March 27th, 2025. Studies were included if they used ML methods to predict treatment response in patients with emotional disorders. Data were extracted on sample size, type of treatment, predictors used, ML methods, and prediction accuracy. Meta-analytic techniques were used to synthesize findings and identify moderators of prediction accuracy.

**Results:**

Out of 3816 non-duplicate records, 155 studies met inclusion criteria. The overall mean prediction accuracy was 0.76 (95 % CI: 0.74–0.78), and the mean area under the curve was 0.80 indicating good discrimination. The average sensitivity and specificity were 0.73 and 0.75, respectively. Moderator analyses indicated that studies using more robust cross-validation procedures exhibited higher prediction accuracy. Neuroimaging data as predictors were associated with higher accuracy compared to clinical and demographic data. Moreover, results indicated that studies with larger responder rates, as well as those that did not correct for imbalances in outcome rates, were associated with higher prediction accuracy.

**Conclusions:**

ML methods show promise in predicting treatment response for emotional disorders, with varying degrees of accuracy depending on the type of predictors used and the rigor of methodological procedures implemented. Future research should focus on improving methodological integrity and exploring the integration of multimodal data to enhance prediction accuracy.

## 1. Introduction

Emotional disorders, such as depression and anxiety, are among the most prevalent and disabling conditions worldwide. These disorders not only eventuate in significant personal suffering and impairment, but also impose a considerable economic and systemic burden on healthcare systems. Traditional evidence-based treatment approaches– including evidence-based psychotherapies (e.g., cognitive behavioral therapy (CBT)), pharmacotherapies, neuromodulation, etc.– have proven efficacious for many patients. That notwithstanding, a substantial proportion of individuals fail to achieve adequate response to these interventions, underscoring the need for more personalized approaches to treatment planning (e.g., identifying what intervention or treatment strategy a patient should receive at a given time on the basis of individual differences in symptom, environmental, lifestyle, genetic, or other characteristics). Response to “gold-standard” evidence-based therapy is modest at best (response rates = 49.5 % – 65.2 %), and patients often continue to suffer significant disease and economic burden (Lemmens et al., 2019; Loerinc et al., 2015).

A primary reason for the unsatisfactory progress in improving treatment for emotional disorders is our inability to accurately predict treatment response. The advent and adoption of machine learning (ML) in medicine may prove profitable for addressing this need. Recent proposals for improving personalized predictions in psychology have illustrated how ML is particularly suited to optimizing prediction, as opposed to traditional statistical frameworks that focus on explanation and mechanistic knowledge regarding potentially causal relationships between psychological constructs (Yarkoni and Westfall, 2017). A salient advantage of certain ML algorithms, such as support vector machines or neural networks, consists in their ability to better capture nonlinear and complex patterns underlying a given data structure (Kuhn and Johnson, 2013; Pigoni et al., 2024; Winter et al., 2024). This is especially germane to psychological and mental health data, which has been posited to exhibit substantial nonlinear, dynamic structures (Hofmann and Curtiss, 2018; Hofmann, Curtiss and Hayes, 2020). In the context of emotional disorders, predictive modeling procedures such as ML can better facilitate precision medicine predictions of individual responses to various treatments, thereby enabling more targeted and effective interventions (Curtiss et al., 2024). ML’s capability to model individual differences is particularly apposite given the heterogeneous nature of emotional disorders, for which factors influencing treatment response can vary widely among individuals.

Recent studies have explored the use of ML to predict treatment outcomes in emotional disorders, utilizing diverse types of data, including clinical, demographic, genetic, and neuroimaging information. Notwithstanding the relative proliferation of such applied ML studies, several challenges remain. Extant studies have demonstrated varying degrees of success, with some reporting high prediction accuracies while others have yielded more modest results (Curtiss et al., 2024; Sajjadian et al., 2021). The variability in these findings highlights the importance of understanding the conditions under which ML models perform best and identifying the key moderating factors that contribute to accurate treatment response predictions. One principal issue is the relative diversity of ML methodological decisions represented in the applied literature, ranging from disparities in pre-processing procedures, management of class imbalances in the outcome data (i.e., responders versus non-responders), modalities of the features, cross-validation procedures, and innumerable other factors (Lee et al., 2018; Sajjadian et al., 2021). In light of the lack of standardization of methodologies across studies, which is essential for comparing results and drawing generalizable conclusions, a comprehensive survey of current practices and their impact on prediction accuracy is imperative.

The current meta-analysis constitutes the first comprehensive review and quantitative synthesis of existing research on the use of ML to predict treatment response in emotional disorders with a specific focus on identifying moderating factors that influence prediction accuracy. Although prior reviews have examined the potential for ML to improve personalized treatment strategies (Lee et al., 2018; Sajjadian et al., 2021), relatively little attention has been devoted toward understanding how disparate ML methodologies and study characteristics affect prediction performance across the spectrum of emotional disorders. This, accordingly, can furnish crucial insights into precision medicine treatment of mental health disorders and facilitate the adoption of better practices in applied ML research in psychiatry.

## 2. Methods

### 2.1. Literature search

A comprehensive literature search was conducted across PubMed and PsycINFO databases from January 1st, 2010 to March 27th, 2025. The search terms included combinations of “machine learning,” “deep learning,” “depression,” “anxiety,”, “social anxiety disorder”, “panic disorder”, “phobia”, “agoraphobia”, “social phobia”, “specific phobia”, “obsessive compulsive disorder”, “post traumatic stress disorder”, “body dysmorphic disorder”, “eating disorder”, “anorexia”, “bulimia”, “treatment,”, “therapy”, “psychotherapy”, “cognitive behavior therapy”, “mindfulness”, “dialectical behavior therapy”, “acceptance and commitment therapy”, “exposure therapy”, “medication”, “pharmacotherapy”, and “biofeedback”. The search strategy aimed to capture all relevant studies that used ML methods to predict treatment response in patients with emotional disorders. For full search terms with inclusion of logical operators refer to the Supplementary Material. This analysis was preregistered with the International Prospective Register of Systematic Reviews (PROSPERO) on October 10th, 2023, and was last updated on October 3rd, 2024 (registration number CRD42023469216).

### 2.2. Inclusion and exclusion criteria

Studies were included if they satisfied the following criteria: 1) Participants had a diagnosis of an emotional disorder (anxiety, depression, or related disorders). 2) The study used ML methods to predict binary treatment response (responder vs. non-responder) or remission (remitter vs. non-remitter). Reports examining continuous outcomes were not considered, given that binary performance metrics (e.g., accuracy) could not be derived. 3) The study reported binary prediction accuracy metrics (accuracy or area under the curve; AUC). 4) The study included adequate data partitioning methods for ML, dividing the dataset into appropriate training (and potentially validation) and test sets (e.g., simple split, external validation, cross-validation, nested cross-validation). 5) The study was written in English and published in a peer-review journal.

Studies were excluded if they did not meet these criteria or if they focused on non-human subjects or non-ML predictive methods. The principal requirement for binary data is necessitated by the fact that binary metrics such as accuracy and AUC are able to be meta-analyzed given that they are in a standardized metric. Furthermore, treatment outcome constructs such as response and remission convey widely recognized information to clinicians, thereby being readily interpretable. Conversely, meta-analyzing continuous performance metrics are beset with problems given that the vast majority of such metrics quantify error in the unstandardized units of the original scales (e.g., mean absolute error, root mean square error, etc.), which precludes meaningful synthesis into composite estimates given heterogeneity in symptom instruments.

### 2.3. Data extraction

Data were extracted independently by two reviewers using a standardized procedure, involving collating data according to whether it was a ML performance metric or a potential moderator variable. Discrepancies in whether certain data should be extracted were resolved by a third reviewer. Extracted data included study characteristics (e.g., year of publication, sample size, type of treatment, response type), ML methods (e.g., type of algorithm, validation method, feature selection), predictors used (e.g., clinical, demographic, neuroimaging, genetic), and prediction accuracy metrics.

#### Data Analysis.

All meta-analytic analyses were conducted in R using the package *metafor* (Viechtbauer, 2010) and *mada* (Doebler, 2015). Multilevel random-effects models were employed to account for heterogeneity between studies. Because some studies reported multiple outcomes (e.g., prediction accuracy for more than one ML algorithm, etc.), the data was inherently nested, thereby necessitating the use of a multilevel modeling meta-analytic approach (Cheung, 2014). That is, the assumption of statistical independence, one of the core assumptions of a traditional meta-analysis, is violated when dependency exists between effect sizes (Harrer et al., 2021). Such statistical dependence, when modeled improperly, can eventuate in reduced heterogeneity, which may prompt overly optimistic estimations of positive results (Harrer et al., 2021). Thus, a multilevel modeling approach was adopted to address dependency that would result from nesting and dependency in outcomes within some of the studies. Consistent with recommendations and default specifications (Viechtbauer, 2010), the error covariance structure was specified as compound symmetric to account for the correlated data structure.

Classification accuracy was the primary outcome metric. Prior to the meta-analysis, accuracy needed to be transformed into two components: (1) the absolute number of correct classification events (i.e., number of correctly classified responders and non-responders) and (2) the total number of events (i.e., the sample size) (Viechtbauer, 2010). Meta-analyzing results of percentages by themselves proves problematic, and accuracy is expressed as a percentage in individual studies, requiring the need for such transformation into whole events. Consistent with a prior meta-analysis (Lee et al., 2018), the number of correct classification events was calculated for each study by multiplying the reported classification accuracy proportion and sample size (*n*) and rounding the product to the nearest whole number. The two components—number of correctly classified events and total number of events—were treated as the numerator and denominator, respectively, to convert to logit transformed proportions (i.e., log-odds) and, thereby, perform a binomial-normal model for the meta-analysis of proportions (Viechtbauer, 2010). The estimated average log-odds from the meta-analysis were back-transformed using the inverse logit to produce results readily interpretable as a proportion reflecting the overall pooled accuracy (Viechtbauer, 2010). As a complement to the aforementioned procedures, a robust-variance estimation (i.e., Satterthwaite approximation through the clubSandwich package) was also conducted using the robust() argument in *metafor* to account for heteroscedasticity and unmodeled dependence between the errors (Pustejovsky and Tipton, 2018).

Furthermore, AUC, as well as sensitivity and specificity, was calculated and meta-analyzed for studies for which confusion matrix values could be derived (i.e., true positive, false positive, true negative, and false negative values). Consistent with the gold-standard procedures codified in the *mada* package (Doebler, 2015), a bivariate normal model was estimated for the logit-transformed pairs of sensitivities and false positive rates to produce a meta-analyzed AUC value (Arends et al., 2008; Reitsma et al., 2005). In accordance with proposed guidelines (Hosmer and Lemeshow, 2000), AUC values approaching 0.5 suggest no discrimination, whereas values between 0.7 and 0.8 suggest acceptable discrimination, values between 0.8 and 0.9 are considered excellent, and values greater than 0.9 reflect outstanding discrimination.

Meta-regressions models were conducted to examine potential moderators of prediction accuracy, including proportion of responders, age, sample size, treatment duration, preprocessing for imbalanced outcomes, feature type, feature selection method, validation method, missing data method, ML algorithm method, use of an ensemble of algorithms, type of treatment, type of outcome, sample race/ethnicity, and percentage of females in sample. For most models a random intercept model was used, but random slopes were included when appropriate should a moderator genuinely vary as a Level 1 variable. Because proportion of responders and, consequently, imbalance correction variables are closely related to sample size, the sample size variable was controlled for in those two models to avoid confounds.

Meta-analytic diagnostics are difficult to obtain from study data organized with a nested format. To calculate meta-analytic diagnostics, a separate meta-analytic model was pursued by conducting within-study averaging for a non-nested equal effects model, which involves extracting the marginal variance-covariance matrix of the estimates based on the multilevel meta-analysis model to obtain comparable results to the original multilevel model. To examine the presence of publication bias, we used the fail-safe N method to determine the number of additional studies with a null result needed to reduce the overall effect size to non-significance (Rosenthal, 1991). Furthermore, the rank correlation test for funnel plot asymmetry was employed to assess the correlation between study accuracy estimates and their variances (Begg and Mazumdar, 1994). Finally, a leave-one-out sensitivity analysis was performed to determine the robustness of the effects.

## 3. Results

### 3.1. Study characteristics

**Fig. 1.**
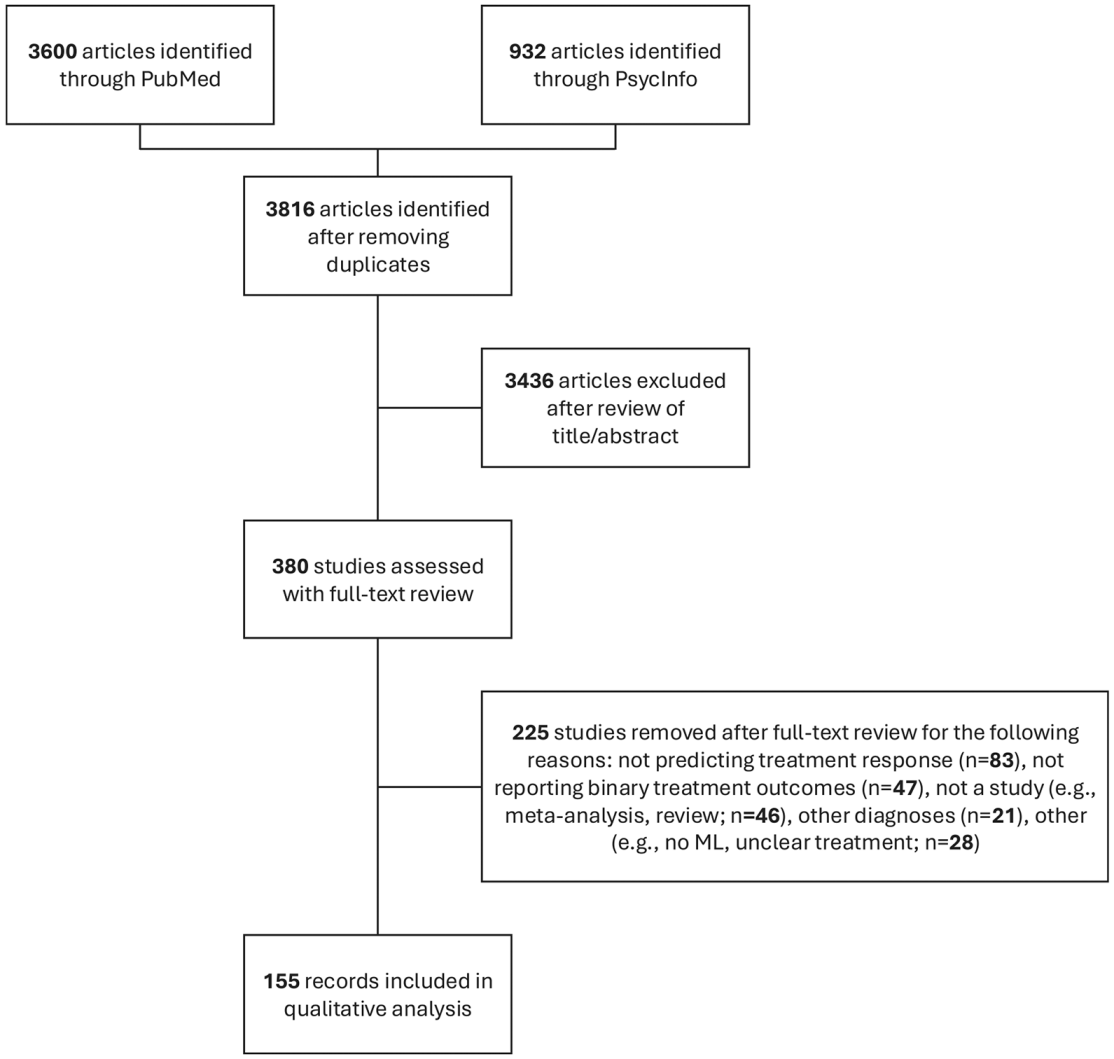
Flow chart.

The initial search yielded 3816 non-duplicate records, of which 155 studies met the inclusion criteria (Fig. 1). The included studies varied in sample size, ranging from 16 to 77,371 participants (mean = 1865, median = 175). The majority of studies focused on pharmacotherapy (*n* = 90), followed by psychotherapies (*n* = 27), bio-stimulation (*n* = 24) and other treatments (e.g., psychedelics, combination therapy and medicine, etc.) (*n* = 14). In terms of different types of imbalance preprocessing procedures, most studies performed no imbalance correction (*n* = 125), and those that did used SMOTE (*n* = 7), down sampling (*n* = 6), up sampling (*n* = 5), weighting (n = 5), and other (n = 7). Regarding type of cross-validation procedure, the most frequently reported were k-fold cross-validation (*n* = 83), simple split (*n* = 55), and nested cross-validation (*n* = 16). For one study, it was undisclosed (Bi et al., 2021), but likely out-of-bag error estimate which is a default of the particular random forest algorithm used in the study. For a fuller understanding of study characteristics, please refer to Table 1.

### 3.2. Prediction accuracy

**Fig. 2.**
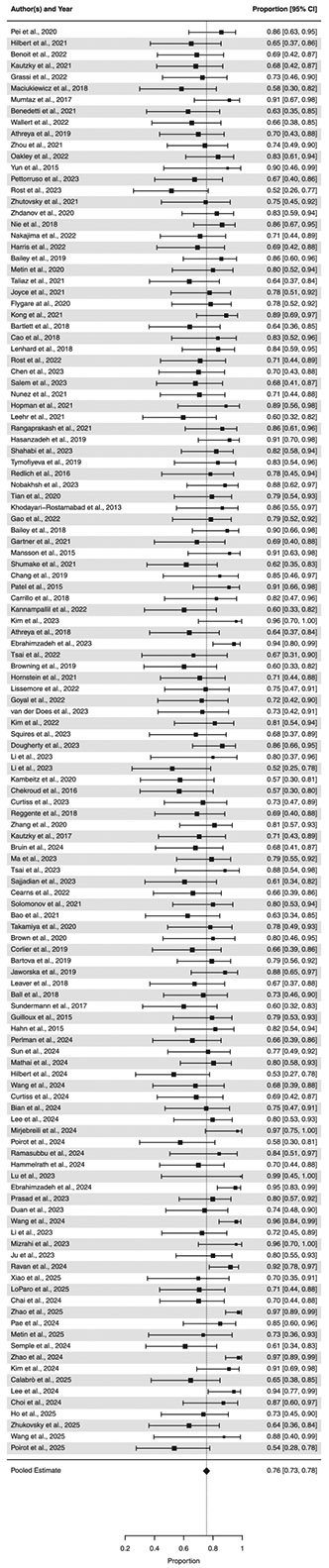
Forest plot. *Notes*: These effects report one pooled effect per study, as determined by the multilevel random-effects model meta-analysis. Please note there might be slight discrepancies in the accuracy values in the Forest Plot and Table 1 due to rounding error. The meta-analyzed results presented in the current figure represent the accuracies calculated from inverse logit back-transformation.

The overall mean prediction accuracy across studies was 0.76 (95 % CI: 0.74–0.78). Moreover, the test for heterogeneity was statistically significant (Q(*df* = 208) = 3919.62, *p* < 0.0001), indicating the presence of potentially meaningful individual differences between study accuracy rates. Refer to Fig. 2 for the full forest plot.

The mean AUC was 0.80 with a range of 0.47 to 0.99 indicating adequate to good discrimination, and the sensitivity and specificity were 0.73 (95 % CI: 0.69–0.77) and 0.75 (95 % CI: 0.72–0.78), respectively.

### 3.3. Moderators of prediction accuracy

**Fig. 3.**
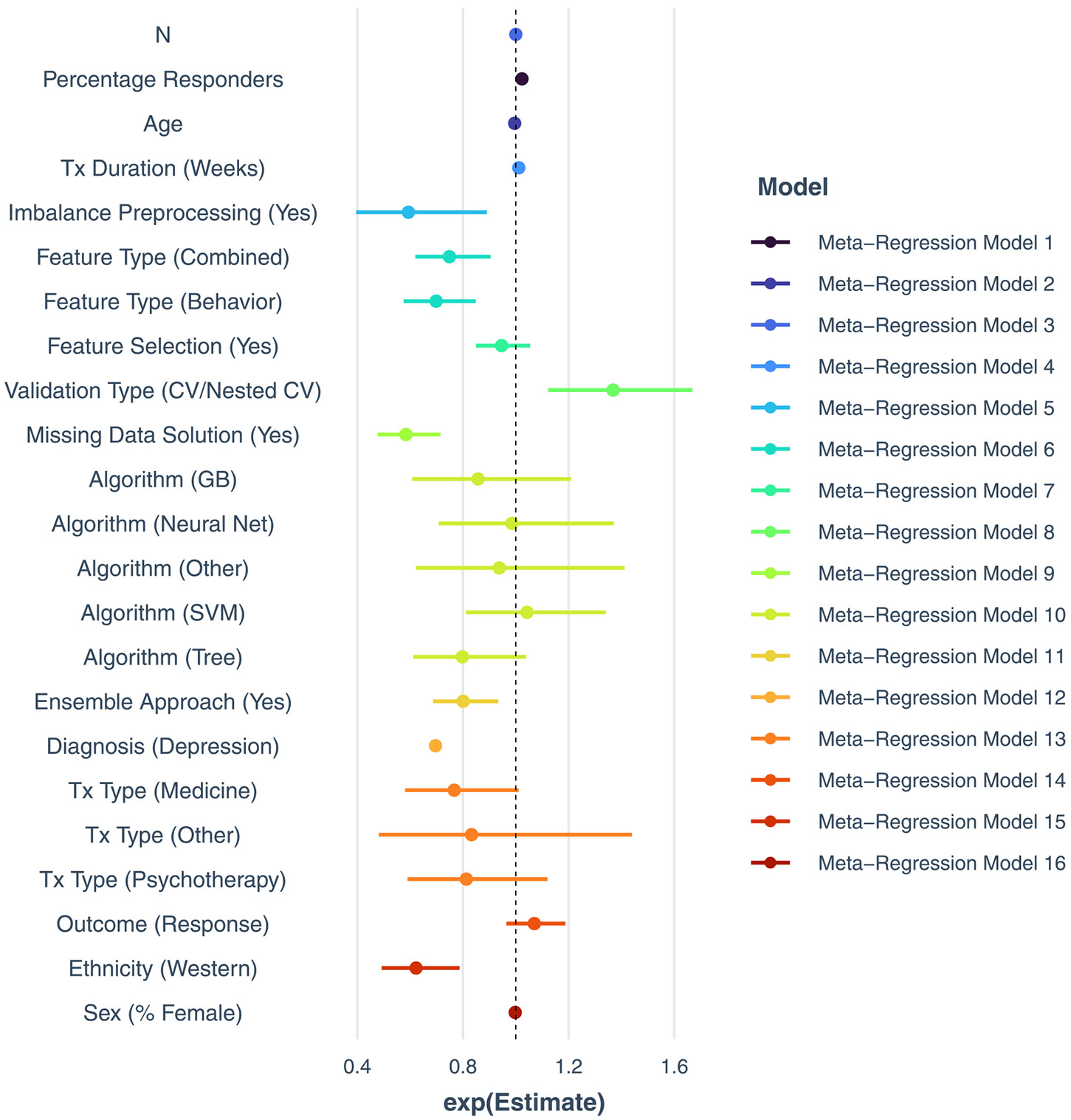
Moderator analyses. *Notes*: These represent exponentiated coefficients to be interpreted as odds ratios, along with their 95 % confidence intervals.

Moderator analyses revealed several factors influencing prediction accuracy (all presented as exponentiated beta coefficients). For a complete description, please refer to Table 2 and Fig. 3 for exponentiated beta coefficients of all meta-regression models. Salient moderators of interest include certain ML methodological properties and clinical characteristics. Larger percentage of responders (β = 1.02, *p* < 0.001) was significantly associated with higher prediction accuracy. The use of imbalance preprocessing (β = 0.59, *p* < 0.05) and addressing missing data (β = 0.58, p < 0.001) were associated with more conservative accuracy estimates. Regression-based ML algorithms (e.g., elastic net) performed comparably to most other sophisticated algorithms (*p*’s > 0.05). Of note, a supplementary analysis indicated that there may be a significant interaction effect between sample size and the comparison between neural networks and regression (*b* = 1.0008, *p* < 0.001). That is, neural network algorithms are associated with greater prediction accuracy under conditions of increased sample sizes (see Supplementary Section). Cross-validation procedures such as k-fold and nested cross-validation were associated with greater accuracy than simple split procedures (β = 1.37, *p* < 0.01).

Treatment type did not significantly impact accuracy (*p*’s > 0.05), suggesting that the quality of the data and validation methods are more critical determinants. That notwithstanding, studies with depressed patient samples exhibited lower prediction accuracy relative to those with non-depressed patient samples (β = 0.70, *p* < 0.001). Length of treatment was modestly and significantly associated with higher prediction accuracies (β = 1.01, *p* < 0.01). Also, in terms of ML features, studies using behavioral predictors (β = 0.75, p < 0.01) or combinations of behavioral and biological predictors (β = 0.70, p < 0.001) exhibited lower prediction accuracies than those using solely biological features (e.g., neuroimaging data).

### 3.4. Meta-analysis diagnostics and robust estimation

The fail-safe N estimate using Viechtbauer’s General Approach was 12,596, which is larger than the 5 k + 10 recommendation indicating that the observed studies are robust to publication bias due to the file drawer problem (Viechtbauer, 2024). That notwithstanding, results of the rank correlation test for funnel plot asymmetry were significant (Kendall’s τ = 0.49, *p* < 0.001), indicating potential publication bias such that studies with smaller sample sizes may be more likely to have larger accuracy estimates. As part of a sensitivity analysis to confirm the robustness of the findings, a leave-one-out analysis was conducted to determine whether the results were influenced by any particular study result. The results indicated a range in accuracies between 0.755 and 0.76, suggesting that the leave-one-out range very closely fits the meta-analysis results and, thus, supports the robustness of the estimate.

Finally, to obtain cluster-robust tests and confidence intervals, a robust variance estimation procedure was estimated. The results remained unchanged with an accuracy estimate of 0.76 (95 % CI: 0.74–0.78), which was significant per the Satterthwaite approximation (*t* = 19.70 (*df* = 114.75), *p* < 0.0001).

## 4. Discussion

The current meta-analysis demonstrates that ML methods can predict treatment response in emotional disorders with moderate accuracy. The findings underscore the potential of ML to augment personalized medicine by enabling the identification of individuals who are most likely to benefit from specific treatments. This capability is particularly relevant in the context of emotional disorders, where treatment responses are highly variable and often unpredictable. Namely, predictive modeling may refine personalized medicine by addressing several hitherto unresolved problems such as: 1) providing prognostic information about the probability of success in response to different treatment options, 2) facilitating shared decision making between patients and clinicians (e.g., among those treatments identified as being potentially successful, which one may a patient select on the basis of other medical and life circumstantial factors, etc.), 3) identifying the most salient predictors from large feature sets to develop more parsimonious and easily implementable treatment outcome calculators, 4) optimizing the order and timing of treatments (e.g., guiding JITAI strategies, etc.), and 5) optimizing the dosing of treatments (Chekroud et al., 2021; Wang, Ouyang, et al., 2024).

Although this meta-analysis provides support for overall optimism regarding the utility of ML in predicting treatment response, results of the moderator analyses reveal potential complicating factors that underscore the need for circumspection. Notably, increases in performance accuracy were significantly predicted by higher responder rates and a lack of addressing outcome class imbalance. This suggests that successful instances of ML prediction merely may be a consequence of imbalances in responder status. When imbalances in binary outcomes occur, ML can exploit this trivial feature of a dataset, and high levels of accuracy can be obtained just by being biased in favor of over-representing the majority class in its predictions (i.e., being a responder instead of a non-responder) (Kuhn and Johnson, 2013). Furthermore, studies that did not account for class imbalances with corrective preprocessing strategies such as SMOTE, down sampling, or up sampling were more likely to achieve higher prediction accuracies, which again may reflect a bias for over-representing the majority class. ML studies that did leverage data sampling preprocessing procedures yielded a more conservative prediction accuracy. Thus, the success of ML in predicting treatment response needs to be construed with caution, given the potential consequences of class imbalances.

The higher accuracy observed in studies utilizing neuroimaging and biological data as predictors suggests that such data may provide valuable insights into the neural mechanisms underlying treatment response (Doehrmann et al., 2013; Gabrieli et al., 2015). Neuroimaging especially can capture complex brain patterns and structural abnormalities that may be associated with how individuals respond to various treatments. Moreover, neuroimaging techniques can elucidate potential biomarkers, which can be employed to define novel subtypes of disorders that might be valuable in determining which individuals are optimal candidates for targeted therapies (Drysdale et al., 2017). However, the integration of neuroimaging data into clinical practice remains a challenge due to high costs and the need for specialized equipment and expertise (Gabrieli et al., 2015). Another challenge of models validated on neuroimaging and biological data is the limited extent to which they generalize to new samples (i.e., the bias-variance tradeoff). Given the considerable heterogeneity in neuroimaging preprocessing procedures and types of neuroimaging data (e.g., region of interest vs. whole brain, task activation vs. resting state, etc.), such diversity in methodologies may impede generalization of predictions in new contexts.

Another important finding is the influence of ML methods (validation type, algorithm type, ensemble usage, and missing data) on prediction accuracy. Studies employing robust validation methods, such as k-fold cross-validation and nested cross-validation rather than simple split, tended to report higher accuracies. Additionally, in terms of algorithm type, the use of simpler regression-based algorithms (e.g., elastic net, regularized regression, etc.) were associated with comparable accuracies as studies employing more sophisticated algorithms (e.g., neural net, SVM, tree-based approaches, etc.). That notwithstanding, neural network algorithms are associated with greater prediction accuracy under conditions of increased sample sizes (see Supplementary Materials). This is perhaps not entirely surprising, given that neural networks and deep learning algorithms typically require large sample sizes (Kuhn and Johnson, 2013). Moreover, lower prediction accuracy was also associated with studies that used ensemble approaches or addressed missing data appropriately (e.g., imputation approaches, etc.). This may indicate that certain methodological decisions may yield more conservative ML prediction estimates, highlighting the importance of methodological rigor in ML research. Future studies should prioritize the use of large, well-characterized datasets and robust validation techniques to ensure the reliability and generalizability of ML models.

Certain clinical and demographic characteristics were also important in influencing ML prediction accuracy of treatment response. Specifically, studies using Western samples, as well as those including patients with depression, evidenced lower prediction accuracy. Although there is no obvious explanation for these findings, it has been acknowledged that prediction of treatment outcomes in depression has generally demonstrated small effects with little success in generalizing clinical prediction models in external populations (Gillett et al., 2020).

### 4.1. Challenges and future directions

Despite these promising findings, there is a need for further research to address existing gaps and challenges. One key area is the standardization of addressing class imbalances in studies predicting treatment response with machine learning. It is of paramount importance for researchers to consider the insidious influence of class imbalance, which may bias ML algorithms to always predict the majority class and, thereby, obtain good classification accuracy without adequately distinguishing between the responder and non-responder outcome classes. Corrective data sampling procedures (e.g., SMOTE) may be advisable to mitigate this concern and facilitate a more balanced assessment of how well ML can actually predict treatment outcomes (Abd Elrahman and Abraham, 2013). Relatedly, future studies should consider more rigorous cross-validation procedures for assessing model performance in test data, such as the implementation of nested cross-validation, which involves two layers of cross-validation for both validating hyperparameters and testing the final model on unseen data. Another challenge is related to the standardization of outcome measures and definitions of treatment response across studies. The variability in these measures makes it difficult to compare results and draw definitive conclusions. Establishing standardized criteria for treatment response and outcome assessment would facilitate more meaningful comparisons and meta-analyses.

Another critical area for future research is the exploration of multimodal data integration. Combining clinical, demographic, genetic, and neuroimaging data could enhance the predictive power of ML models by providing a more comprehensive understanding of the factors influencing treatment response. Multimodal data integration would furthermore profit from judicious feature engineering pipelines. Feature engineering, particularly variable filtering, can enhance ML performance by eliminating irrelevant or redundant features that can introduce noise into the model. This process allows the model to concentrate on the most impactful variables, improving accuracy, reducing overfitting, and yielding a more parsimonious feature set for predicting outcomes (Kuhn and Johnson, 2019), although such procedures should only be undertaken on the non-testing subsets (i.e., training/validation) of the data to preclude data leakage. Additionally, advancements in ML algorithms and computational techniques, such as time-series forecasting, hold promise for further improving prediction accuracy and clinical applicability at the individual level by exploiting personalized symptom dynamics that unfold over time.

A final limitation meriting discussion is that the meta-analysis diagnostics may indicate potential publication bias, such that studies with smaller sample sizes may be more likely to have larger accuracy estimates. This attests to the need for future research to leverage larger samples to provide more reliable and valid appraisals of ML prediction performance.

Future research should aim to standardize methodologies for ML-based prediction of treatment response, including the use of consistent corrective sampling procedures for imbalanced data and cross-validation methods. Additionally, the integration of multimodal data (e.g., combining clinical, genetic, and neuroimaging data) may further improve prediction accuracy. Large-scale collaborative studies are needed to validate and refine ML models in diverse patient populations.

## 5. Conclusion

Machine learning holds promise for predicting treatment response in emotional disorders, with potential applications in personalized medicine. While current prediction accuracies are optimistically moderate, advancements in data integration and methodological rigor may enhance the utility of ML in clinical practice. This meta-analysis provides a comprehensive overview of the current state of ML-based prediction in emotional disorders and highlights key areas for future research.

## Supplementary Material

Supplement Methods and Results

Supplement Table

Supplement Fig 1

## Figures and Tables

**Table 1:** Study characteristics.

Study ID	Author	Year	Accuracy	AUC	N	Diagnosis	Treatment	ML Algorithm	Feature Type	Validation Type
1	Pei et al.	2020	0.86		98	MDD	Medicine	SVM	Combined	cross validation
2	Aderka et al.	2021		0.50	1514	MDD	Psychotherapy	SVM	Behavioral	simple split
3	Hilbert et al.	2021	0.65	0.72	458	OCD	Psychotherapy	Random Forest	Behavioral	simple split
4	Benoit et al.	2022	0.69		3776	MDD	Medicine	SVM	Combined	simple split
5	Kautzky et al.	2021	0.69		504	MDD	Medicine	Random Forest	Behavioral	cross validation
5	Kautzky et al.	2021	0.62		504	MDD	Medicine	Random Forest	Behavioral	cross validation
5	Kautzky et al.	2021	0.69		204	MDD	Medicine	Random Forest	Behavioral	cross validation
5	Kautzky et al.	2021	0.69		131	MDD	Medicine	Random Forest	Behavioral	cross validation
5	Kautzky et al.	2021	0.82		121	MDD	Medicine	Random Forest	Behavioral	cross validation
5	Kautzky et al.	2021	0.81		127	MDD	Medicine	Random Forest	Behavioral	cross validation
6	Rosellini et al.	2023		0.61	1210	MEmD	Psychotherapy	Super Learner	Behavioral	simple split
6	Rosellini et al.	2023		0.71	1210	MEmD	Psychotherapy	Super Learner	Behavioral	simple split
6	Rosellini et al.	2023		0.73	1210	MEmD	Psychotherapy	Super Learner	Behavioral	simple split
7	Grassi et al.	2022	0.72	0.78	287	OCD	Combined (Unspecified)	GB	Behavioral	simple split
8	Maciukiewicz et al.	2018	0.66		186	MDD	Medicine	SVM	Combined	nested cross validation
8	Maciukiewicz et al.	2018	0.52		186	MDD	Medicine	SVM	Combined	nested cross validation
9	Mumtaz et al.	2017	0.92		34	MDD	Medicine	Logistic Regression	Psychophys	cross validation
10	Hoogendoorn et al.	2016		0.78	69	SAD	Psychotherapy	Random Forest	Behavioral	cross validation
11	Benedetti et al.	2021	0.63	0.75	108	TRMDD	Medicine	Elastic Net	Combined	nested cross validation
12	Wallert et al.	2022	0.66		894	MDD	Psychotherapy	Random Forest	Combined	simple split
13	Athreya et al.	2019	0.69		326	MDD	Medicine	Random Forest	Combined	simple split
13	Athreya et al.	2019	0.66		539	MDD	Medicine	Random Forest	Combined	simple split
13	Athreya et al.	2019	0.77		206	MDD	Medicine	Random Forest	Combined	simple split
13	Athreya et al.	2019	0.75		357	MDD	Medicine	Random Forest	Combined	simple split
13	Athreya et al.	2019	0.75		326	MDD	Medicine	Random Forest	Combined	simple split
13	Athreya et al.	2019	0.66		539	MDD	Medicine	Random Forest	Combined	simple split
13	Athreya et al.	2019	0.76		206	MDD	Medicine	Random Forest	Combined	simple split
13	Athreya et al.	2019	0.74		357	MDD	Medicine	Random Forest	Combined	simple split
14	Zhou et al.	2021	0.74	0.73	400	MDD	Medicine	SVM	Behavioral	cross validation
15	Held et al.	2022		0.47	432	PTSD	Psychotherapy	GB	Behavioral	nested cross validation
16	Oakley et al.	2022	0.84	0.84	228	MDD	Medicine	SVM	Psychophys	cross validation
16	Oakley et al.	2022	0.83	0.82	228	MDD	Medicine	SVM	Psychophys	cross validation
17	Yun et al.	2015	0.89		56	OCD	Medicine	SVM	Neuroimaging	simple split
18	Pettorruso et al.	2023	0.66		149	TRMDD	Psychedelic	Random Forest	Behavioral	cross validation
18	Pettorruso et al.	2023	0.69		149	TRMDD	Psychedelic	Random Forest	Behavioral	cross validation
19	Rost et al.	2023	0.60		1102	MDD	Medicine	Elastic Net	Behavioral	simple split
19	Rost et al.	2023	0.41		1102	MDD	Medicine	Elastic Net	Combined	simple split
19	Rost et al.	2023	0.54		1102	MDD	Medicine	Elastic Net	Combined	simple split
20	Zhutovsky et al.	2021	0.76	0.82	40	PTSD	Psychotherapy	SVM	Neuroimaging	cross validation
21	Zhdanov et al.	2020	0.82		115	MDD	Medicine	SVM	Psychophys	cross validation
22	Nie et al.	2018	0.86	0.73	2679	MDD	Medicine	Random Forest	Behavioral	simple split
22	Nie et al.	2018	0.86		3007	MDD	Medicine	Random Forest	Behavioral	simple split
23	Nakajima et al.	2022	0.71		177	MDD_BD	BS	GB	Behavioral	cross validation
24	Harris et al.	2022	0.70		144	MDD	Medicine	Random Forest	Neuroimaging	cross validation
25	Bailey et al.	2019	0.87		42	MDD	BS	SVM	Psychophys	cross validation
26	Metin et al.	2020	0.80		50	OCD	BS	Neural Network	Psychophys	cross validation
27	Taliaz et al.	2021	0.60		1829	MDD	Medicine	SVM	Combined	simple split
27	Taliaz et al.	2021	0.75		1829	MDD	Medicine	SVM	Combined	simple split
27	Taliaz et al.	2021	0.75		1829	MDD	Medicine	SVM	Combined	simple split
27	Taliaz et al.	2021	0.68		1829	MDD	Medicine	SVM	Combined	simple split
28	Joyce et al.	2021	0.78	0.86	348	MDD	Medicine	Penalized Regression	Combined	simple split
29	Flygare et al.	2020	0.78	0.78	88	BDD	Psychotherapy	Random Forest	Behavioral	cross validation
30	Perlis	2013		0.72	2555	MDD	Medicine	Logistic Regression	Behavioral	simple split
31	Kong et al.	2021	0.90		82	MDD	Medicine	CNN	Neuroimaging	cross validation
32	Bartlett et al.	2018	0.64	0.59	184	MDD	Medicine	Random Forest	Combined	cross validation
33	Cao et al.	2018	0.83	0.90	24	MDD	BS	Support Vector Regression (SVR)	Neuroimaging	cross validation
34	Lenhard et al.	2018	0.83		61	OCD	Psychotherapy	Linear Model	Behavioral	cross validation
34	Lenhard et al.	2018	0.83		61	OCD	Psychotherapy	Linear Model	Behavioral	simple split
35	Rost et al.	2022	0.71		1022	MDD	Medicine	Elastic Net	Behavioral	simple split
36	Chen et al.	2023	0.70		291	MDD	Medicine	Random Forest	Combined	cross validation
37	Salem et al.	2023	0.66	0.73	1486	MDD	Medicine	Random Forest	Behavioral	cross validation
37	Salem et al.	2023	0.71	0.78	534	MDD	Medicine	Random Forest	Behavioral	cross validation
37	Salem et al.	2023	0.85	0.84	104	MDD	Medicine	Random Forest	Behavioral	cross validation
38	Nunez et al.	2021	0.65	0.69	3024	MDD	Medicine	Random Forest	Behavioral	simple split
38	Nunez et al.	2021	0.77	0.80	3024	MDD	Medicine	Logistic Regression	Behavioral	simple split
39	Hopman et al.	2021	0.89	1.00	61	TRMDD	BS	SVM	Neuroimaging	simple split
40	Leehr et al.	2021	0.60		171	SP	Psychotherapy	Random Forest	Behavioral	cross validation
41	Rangaprakash et al.	2021	0.86		44	OCD	Psychotherapy	SVM	Neuroimaging	cross validation
42	Hasanzadeh et al.	2019	0.91		46	MDD	BS	KNN	Psychophys	cross validation
43	Shahabi et al.	2023	0.82	0.83	170	MDD	BS	CNN	Psychophys	simple split
44	Tymofiyeva et al.	2019	0.83		30	MDD	Psychotherapy	J48 pruned tree classifier	Combined	cross validation
45	Redlich et al.	2016	0.78		23	MDD	BS	SVM	Neuroimaging	cross validation
46	Nobakhsh et al.	2023	0.90		34	MDD	BS	SVM	Psychophys	cross validation
47	Iniesta et al.	2016		0.72	793	MDD	Medicine	Elastic Net	Behavioral	cross validation
47	Iniesta et al.	2016		0.72	465	MDD	Medicine	Elastic Net	Behavioral	cross validation
47	Iniesta et al.	2016		0.70	328	MDD	Medicine	Elastic Net	Behavioral	cross validation
48	Zandvakili et al.	2019		0.83	35	MDD_PTSD	BS	LASSO	Psychophys	cross validation
48	Zandvakili et al.	2019		0.71	35	MDD_PTSD	BS	LASSO	Psychophys	cross validation
49	Tian et al.	2020	0.79		106	MDD	Medicine	SVM	Neuroimaging	cross validation
50	Khodayari-Rostamabad et al.	2013	0.88		22	MDD	Medicine	SVM	Psychophys	cross validation
51	Gao et al.	2022	0.79	0.83	98	MDD	Medicine	Logistic Regression	Combined	cross validation
52	Bailey et al.	2018	0.91		39	MDD	BS	SVM	Combined	cross validation
53	Gärtner et al.	2021	0.69		71	MAD	BS	SVM	Neuroimaging	cross validation
54	Bone et al.	2021		0.82	77,371	MEmD	Psychotherapy	Logistic Regression	Behavioral	simple split
55	Månsson et al.	2015	0.92	0.91	26	SAD	Psychotherapy	SVM	Neuroimaging	cross validation
56	Shumake et al.	2021	0.62	0.66	1663	MDD	Medicine	Meta Learner	Behavioral	cross validation
56	Shumake et al.	2021	0.63	0.66	1663	MDD	Medicine	Meta Learner	Combined	cross validation
56	Shumake et al.	2021	0.61	0.66	1663	MDD	Medicine	Meta Learner	Combined	cross validation
57	Chang et al.	2019	0.85		121	MDD	Medicine	Neural Network	Combined	simple split
58	Wang et al.	2023		0.71	110	MDD	Medicine	Random Forest	Molecular	cross validation
59	Patel et al.	2015	0.89		33	MDD	Medicine	ADTree	Behavioral	nested cross validation
60	Nguyen et al.	2022		0.62	106	MDD	Medicine	Neural Network	Combined	nested cross validation
60	Nguyen et al.	2022		0.60	106	MDD	Medicine	Neural Network	Combined	nested cross validation
60	Nguyen et al.	2022		0.67	116	MDD	Medicine	Neural Network	Combined	nested cross validation
60	Nguyen et al.	2022		0.65	116	MDD	Medicine	Neural Network	Combined	nested cross validation
60	Nguyen et al.	2022		0.57	37	MDD	Medicine	Neural Network	Combined	nested cross validation
60	Nguyen et al.	2022		0.71	37	MDD	Medicine	Neural Network	Combined	nested cross validation
61	Carrillo et al.	2018	0.85		17	TRMDD	Psychedelic	GNBC	Behavioral	cross validation
62	Bossarte et al.	2023		0.66	658	MDD	Medicine + Psychotherapy	Super Learner	Behavioral	simple split
63	Vetter et al.	2022		0.83	239	MDD	Psychotherapy	GB	Behavioral	simple split
64	Kannampallil et al.	2022	0.58	0.67	235	MDD	Psychotherapy	SVM	Behavioral	simple split
64	Kannampallil et al.	2022	0.62	0.71	235	MDD	Psychotherapy	SVM	Behavioral	simple split
65	Kim et al.	2023	0.94		24	MDD	Medicine	Neural Network	Smart Phone	cross validation
66	Lin et al.	2020		0.83	421	MDD	Medicine	GB	Combined	cross validation
66	Lin et al.	2020		0.81	421	MDD	Medicine	GB	Combined	cross validation
67	Athreya et al.	2018	0.64	0.68	99	MDD	Medicine	Linear Model	Combined	nested cross validation
67	Athreya et al.	2018	0.68	0.78	99	MDD	Medicine	SVM	Combined	nested cross validation
67	Athreya et al.	2018	0.72	0.74	191	MDD	Medicine	SVM	Combined	nested cross validation
67	Athreya et al.	2018	0.54	0.53	191	MDD	Medicine	SVM	Combined	nested cross validation
68	Ebrahimzadeh et al.	2023	0.94		88	MDD	BS	SVM	Psychophys	cross validation
69	Tsai et al.	2022	0.69	0.83	70	MDD	Medicine	Neural Network	Combined	simple split
70	Browning et al.	2019	0.60		296	MDD	Medicine	SVM	Behavioral	simple split
71	Hornstein et al.	2021	0.71	0.60	1236	MEmD	Psychotherapy	Random Forest	Behavioral	simple split
72	Lissemore et al.	2022	0.75		76	MDD	BS + Medicine	SVM	Combined	cross validation
73	Goyal et al.	2022	0.72		189	BED	Medicine	GNBC	Behavioral	simple split
74	van der Does et al.	2023	0.73	0.69	292	MSMI	BS	Logistic Regression	Behavioral	simple split
75	Díaz-Zuluaga et al.	2023		0.89	172	BD	Medicine	CART	Combined	simple split
76	Kim et al.	2022	0.82	0.93	48	PTSD	BS	SVM	Psychophys	cross validation
77	Squires et al.	2023	0.94		61	MDD	BS	Neural Network	Neuroimaging	simple split
77	Squires et al.	2023	0.63		133	MDD	BS	Neural Network	Behavioral	simple split
78	Grzenda et al.	2021		0.79	67	MDD	Medicine + Psychotherapy	Random Forest	Behavioral	cross validation
78	Grzenda et al.	2021		0.81	67	MDD	Medicine + Psychotherapy	SVM	Neuroimaging	cross validation
78	Grzenda et al.	2021		0.84	67	MDD	Medicine + Psychotherapy	Random Forest	Combined	cross validation
79	Coley et al.	2021		0.61	5554	MDD	Psychotherapy	Random Forest	Behavioral	simple split
80	Dougherty et al.	2023	0.85	0.88	101	TRMDD	Psychedelic	Logistic Regression	Behavioral	cross validation
80	Dougherty et al.	2023	0.88	0.85	90	TRMDD	Psychedelic	Logistic Regression	Behavioral	cross validation
81	Li et al.	2023a	0.82	0.86	40	TRMDD	BS	SVM	Neuroimaging	simple split
82	Li et al.	2023	0.61		163	MDD	BS	SVM	Psychophys	simple split
82	Li et al.	2023	0.46		163	MDD	BS	SVM	Psychophys	simple split
83	Kambeitz et al.	2020	0.65	0.65	94	MDD	BS + Medicine	GB	Behavioral	cross validation
83	Kambeitz et al.	2020	0.60	0.64	91	MDD	BS + Medicine	GB	Behavioral	cross validation
83	Kambeitz et al.	2020	0.43	0.47	60	MDD	BS + Medicine	GB	Behavioral	cross validation
84	Delgadillo et al.	2020		0.59	1435	MDD	Psychotherapy	Elastic Net	Behavioral	simple split
84	Delgadillo et al.	2020		0.65	1435	MDD	Psychotherapy	Elastic Net	Behavioral	simple split
85	Chekroud et al.	2016	0.60		2410	MDD	Medicine	GB	Behavioral	simple split
85	Chekroud et al.	2016	0.60		2410	MDD	Medicine	GB	Behavioral	simple split
85	Chekroud et al.	2016	0.51		2410	MDD	Medicine	GB	Behavioral	simple split
86	Curtiss et al.	2023	0.73	0.77	97	BDD	Medicine	SVM	Behavioral	cross validation
86	Curtiss et al.	2023	0.70	0.75	97	BDD	Medicine	SVM	Behavioral	cross validation
86	Curtiss et al.	2023	0.76	0.79	97	BDD	Medicine	SVM	Behavioral	cross validation
87	Reggente et al.	2018	0.70		42	OCD	Psychotherapy	SVM	Neuroimaging	cross validation
87	Reggente et al.	2018	0.68		42	OCD	Psychotherapy	SVM	Neuroimaging	cross validation
88	Forrest et al.	2023		0.59	191	BED	Psychotherapy	Elastic Net	Combined	cross validation
89	Zhang et al.	2020	0.76		606	MDD	Medicine	SVM	Behavioral	simple split
89	Zhang et al.	2020	0.88		606	MDD	Medicine	SVM	Combined	simple split
90	Kautzky et al.	2017	0.71		552	MDD	Medicine	Random Forest	Behavioral	simple split
91	Bruin et al.	2024	0.64	0.70	189	MDD	BS	SVM	Combined	cross validation
91	Bruin et al.	2024	0.77	0.80	109	MDD	BS	SVM	Combined	cross validation
92	Ma et al.	2023	0.65	0.78	85	MDD	Medicine	SVM	Neuroimaging	cross validation
92	Ma et al.	2023	0.73	0.80	90	MDD	Medicine	SVM	Neuroimaging	cross validation
92	Ma et al.	2023	0.79	0.80	121	MDD	Medicine	SVM	Neuroimaging	cross validation
92	Ma et al.	2023	0.89	0.95	85	MDD	Medicine	SVM	Neuroimaging	cross validation
92	Ma et al.	2023	0.87	0.94	90	MDD	Medicine	SVM	Neuroimaging	cross validation
92	Ma et al.	2023	0.85	0.89	121	MDD	Medicine	SVM	Neuroimaging	cross validation
93	Tsai et al.	2023	0.91	0.95	58	MDD	BS	KNN	Psychophys	simple split
93	Tsai et al.	2023	0.89	0.92	55	MDD	Medicine	KNN	Psychophys	simple split
94	Sajjadian et al.	2023	0.62	0.64	192	MDD	Medicine	SVM	Combined	nested cross validation
94	Sajjadian et al.	2023	0.61	0.61	192	MDD	Medicine	Random Forest	Molecular	nested cross validation
94	Sajjadian et al.	2023	0.59	0.63	192	MDD	Medicine	Random Forest	Combined	nested cross validation
95	Cearns et al.	2022	0.63	0.66	1034	BD	Medicine	Random Forest	Behavioral	simple split
95	Cearns et al.	2022	0.70	0.70	1034	BD	Medicine	Random Forest	Combined	simple split
96	Puac-Polanco et al.	2023		0.66	660	MDD	Medicine	Super Learner	Behavioral	simple split
97	Ziobrowski et al.	2023		0.65	807	MDD	Psychotherapy	LASSO	Behavioral	simple split
98	Solomonov et al.	2021	0.80		221	MDD	Psychotherapy	Random Forest	Behavioral	simple split
99	Bao et al.	2021	0.63		83	MDD	Psychedelic	Logistic Regression	Combined	nested cross validation
100	Bi et al.	2021		0.77	534	MDD	Medicine	Random Forest	Combined	undisclosed*
100	Bi et al.	2021		0.75	534	MDD	Medicine	Random Forest	Combined	udisclosed*
101	Takamiya et al.	2020	0.70		27	MDD	BS	SVM	Behavioral	cross validation
101	Takamiya et al.	2020	0.93		27	MDD	BS	SVM	Combined	cross validation
102	Brown et al.	2020	0.80		20	MDD_BD	BS	GNBC	Neuroimaging	cross validation
103	Qi et al.	2020		0.57	138	MDD	Medicine	GB	Combined	simple split
104	Corlier et al.	2019	0.62	0.53	109	MDD	BS	Elastic Net	Neuroimaging	cross validation
104	Corlier et al.	2019	0.65	0.58	109	MDD	BS	Elastic Net	Neuroimaging	cross validation
104	Corlier et al.	2019	0.69	0.66	109	MDD	BS	Elastic Net	Neuroimaging	cross validation
104	Corlier et al.	2019	0.77	0.76	68	MDD	BS	Elastic Net	Neuroimaging	cross validation
105	Bartova et al.	2019	0.85		2762	MDD	Medicine	Random Forest	Combined	simple split
105	Bartova et al.	2019	0.75		2762	MDD	Medicine	Random Forest	Combined	simple split
106	Jaworska et al.	2019	0.88	0.90	51	MDD	Medicine	Random Forest	Combined	cross validation
107	Lin et al.	2018		0.82	421	MDD	Medicine	Neural Network	Combined	cross validation
107	Lin et al.	2018		0.81	421	MDD	Medicine	Neural Network	Combined	cross validation
108	Leaver et al.	2018	0.68		46	MDD	BS	SVM	Neuroimaging	cross validation
109	Ball et al.	2014	0.69		48	MEmD	Psychotherapy	Random Forest	Behavioral	cross validation
109	Ball et al.	2014	0.79		48	MEmD	Psychotherapy	Random Forest	Neuroimaging	cross validation
109	Ball et al.	2014	0.73		48	MEmD	Psychotherapy	Random Forest	Combined	cross validation
110	Sundermann et al.	2017	0.54		59	PD	Psychotherapy	SVM	Neuroimaging	cross validation
110	Sundermann et al.	2017	0.67		54	PD	Psychotherapy	SVM	Neuroimaging	cross validation
111	Guilloux et al.	2015	0.76		34	MDD	Medicine	SVM	Molecular	simple split
111	Guilloux et al.	2015	0.97		34	MDD	Medicine	SVM	Combined	simple split
112	Hahn et al.	2015	0.82		49	PD	Psychotherapy	GPC	Neuroimaging	nested cross validation
113	Perlman et al.	2024	0.66	0.70	5032	MDD	Medicine	Neural Network	Behavioral	simple split
114	Sun et al.	2024	0.77	0.86	86	MDD	BS	Logistic Regression	Neuroimaging	cross validation
115	Mathai et al.	2024	0.80	0.81	11,441	MEmD	Psychedelic	Random Forest	Behavioral	nested cross validation
116	Hilbert et al.	2024	0.51		220	MEmD	Psychotherapy	Meta Learner	Combined	simple split
116	Hilbert et al.	2024	0.56		190	SP	Psychotherapy	Meta Learner	Combined	nested cross validation
117	Wang et al.	2024b	0.68	0.65	225	MDD	Medicine	Meta Learner	Combined	simple split
118	Curtiss et al.	2024	0.76	0.70	239	MDD	Medicine	Super Learner	Behavioral	nested cross validation
118	Curtiss et al.	2024	0.59	0.55	279	MDD	Medicine	Super Learner	Behavioral	nested cross validation
118	Curtiss et al.	2024	0.66	0.53	286	MDD	Medicine	Super Learner	Behavioral	nested cross validation
118	Curtiss et al.	2024	0.71	0.65	85	MDD	Medicine + Psychotherapy	Super Learner	Behavioral	nested cross validation
118	Curtiss et al.	2024	0.80	0.72	62	MDD	Psychotherapy	Super Learner	Behavioral	nested cross validation
118	Curtiss et al.	2024	0.70	0.59	238	MDD	Medicine	Super Learner	Behavioral	nested cross validation
118	Curtiss et al.	2024	0.71	0.60	250	MDD	Medicine	Super Learner	Behavioral	nested cross validation
119	Bian et al.	2024	0.75	0.76	69	MDD	Medicine	GNBC	Combined	nested cross validation
120	Lee et al.	2024a	0.80	0.79	247	MDD	Medicine	GB	Combined	simple split
121	Copa et al.	2024		0.95	16	TRMDD	Psychedelic	GB	Neuroimaging	cross validation
122	Mirjebreili et al.	2024	0.98		30	MDD	Medicine	CNN	Neuroimaging	cross validation
123	Poirot et al.	2024	0.52	0.56	109	MDD	Medicine	GB	Neuroimaging	simple split
123	Poirot et al.	2024	0.64	0.68	109	MDD	Medicine	GB	Neuroimaging	simple split
124	Ramasubbu et al.	2024	0.84		19	MDD_BD	BS	GNBC	Combined	cross validation
125	Hammelrath et al.	2024	0.70	0.77	1591	MDD	Psychotherapy	Random Forest	Behavioral	nested cross validation
126	Xu et al.	2023		0.77	808	MDD	Medicine	GB	Behavioral	nested cross validation
127	Lu et al.	2023	0.99		17	MDD	Medicine	CNN	Psychophys	cross validation
128	Ebrahimzadeh et al.	2024	0.95		106	MDD	BS	SVM	Psychophys	cross validation
129	Prasad et al.	2023	0.78		45,352	MDD	Psychotherapy	Neural Network	Behavioral	simple split
129	Prasad et al.	2023	0.77		45,352	MDD	Psychotherapy	Neural Network	Behavioral	simple split
129	Prasad et al.	2023	0.77		45,352	MDD	Psychotherapy	Neural Network	Behavioral	simple split
129	Prasad et al.	2023	0.86		45,756	Anxiety	Psychotherapy	Neural Network	Behavioral	simple split
129	Prasad et al.	2023	0.83		45,756	Anxiety	Psychotherapy	Neural Network	Behavioral	simple split
129	Prasad et al.	2023	0.82		45,756	Anxiety	Psychotherapy	Neural Network	Behavioral	simple split
130	Duan et al.	2023	0.74	0.74	198	MDD_BD	Medicine	Neural Network	Neuroimaging	cross validation
131	Wang et al.	2024c	0.96	1.00	98	MDD	Medicine	SVM	Combined	cross validation
132	Christ et al.	2023		0.64	235	PTSD	Psychotherapy	GB	Behavioral	nested cross validation
132	Christ et al.	2023		0.57	465	PTSD	Psychotherapy	SVM	Behavioral	nested cross validation
133	Li et al.	2023b	0.74		62	MDD	BS	Random Forest	Psychophys	cross validation
133	Li et al.	2023	0.71		69	MDD	BS	Random Forest	Psychophys	cross validation
134	Mizrahi et al.	2023	0.96		24	BD	Medicine	SVM	Genetic	nested cross validation
135	Ju et al.	2023	0.80		110	MDD	Medicine	SVM	Neuroimaging	cross validation
136	Carr et al.	2025		0.818	214	MDD	Medicine	Logistic Regression	Combined	cross validation
136	Carr et al.	2025		0.834	196	MDD	Medicine	Logistic Regression	Combined	cross validation
136	Carr et al.	2025		0.844	714	MDD	Medicine	Logistic Regression	Combined	cross validation
137	Ravan et al.	2024	0.91	0.91	105	MDD	Medicine	CNN	Psychophys	cross validation
137	Ravan et al.	2024	0.95	0.95	119	MDD	Medicine	CNN	Psychophys	cross validation
137	Ravan et al.	2024	0.87	0.90	25	MDD	Medicine	CNN	Psychophys	cross validation
138	Bertie et al.	2024		0.69	2114	Anxiety	Psychotherapy	Neural Network	Behavioral	cross validation
138	Bertie et al.	2024		0.68	2114	Anxiety	Psychotherapy	Neural Network	Behavioral	cross validation
139	Xiao et al.	2025	0.69		20	BD	BS	Neural Network	Psychophys	cross validation
140	LoParo et al.	2025	0.61		248	MDD	Medicine	Linear Model	Behavioral	cross validation
140	LoParo et al.	2025	0.81		248	MDD	Medicine	Linear Model	Behavioral	cross validation
140	LoParo et al.	2025	0.73		248	MDD	Psychotherapy	Linear Model	Behavioral	cross validation
141	Chai et al.	2024	0.70	0.76	4067	Anxiety	Psychotherapy	GB	Behavioral	simple split
142	Zhao et al.	2025	0.97	0.99	117	MDD	BS	SVM	Combined	cross validation
143	Pae et al.	2024	0.85	0.84	53	PD	Medicine	SVM	Neuroimaging	cross validation
144	Metin et al.	2025	0.73	0.78	120	MDD	Medicine	CNN	Psychophys	simple split
145	Semple et al.	2024	0.61	0.65	2074	MDD	BS	SVM	Behavioral	cross validation
146	Zhao et al.	2024	0.97	0.96	117	MDD	BS	Adaboost	Psychophys	cross validation
147	Kim et al.	2024	0.91	0.91	98	MDD	Medicine	CNN	Blood	cross validation
148	Calabrò et al.	2025	0.65		820	MDD	Medicine	Tree Classifier	Combined	cross validation
149	Lee et al.	2024b	0.94		52	MDD	Medicine	SVM	Combined	cross validation
150	Marrero-Polanco et al.	2025		0.70	505	BD	Medicine	GB	Behavioral	simple split
150	Marrero-Polanco et al.	2025		0.75	505	BD	Medicine	Penalized Regression	Behavioral	simple split
150	Marrero-Polanco et al.	2025		0.73	505	BD	Medicine	GB	Behavioral	simple split
151	Choi et al.	2024	0.87	0.83	31	MDD	Medicine	Linear Model	Psychophys	cross validation
152	Ho et al.	2025	0.73	0.77	64	MDD	Medicine	GNBC	Neuroimaging	cross validation
153	Zhukovsky et al.	2025	0.63	0.62	363	MDD	Medicine	Elastic Net	Combined	simple split
153	Zhukovsky et al.	2025	0.65	0.67	363	MDD	Medicine	Elastic Net	Combined	simple split
153	Zhukovsky et al.	2025	0.64	0.66	363	MDD	Medicine	Elastic Net	Combined	simple split
154	Wang et al.	2025	0.88		30	MDD	Medicine	SVM	Psychophys	simple split
155	Poirot et al.	2025	0.56		262	MDD	Medicine	SVM	Neuroimaging	cross validation
155	Poirot et al.	2025	0.54		262	MDD	Medicine	GB	Neuroimaging	cross validation
155	Poirot et al.	2025	0.51		262	MDD	Medicine	Neural Network	Neuroimaging	cross validation

*Note:* *For one study (Bi et al., 2021), the cross-validation procedure was not explicitly detailed, but was likely out-of-bag error estimation, which is a default of the particular random forest algorithm used in that study. The list of abbreviations provided represents various mental health conditions, where MDD stands for Major Depressive Disorder, MEmD indicates Mixed Emotional Disorders, SAD refers to Social Anxiety Disorder, TRMDD denotes Treatment Resistant Major Depressive Disorder, BDD represents Body Dysmorphic Disorder, PD signifies Panic Disorder, SP stands for Specific Phobia, MAD indicates Major Affective Disorder, MDD_BD represents Major Depressive Disorder and Bipolar Disorder, MDD_PTSD denotes Major Depressive Disorder and Post Traumatic Stress Disorder, BED refers to Binge Eating Disorder, MSMI indicates Mixed Severe Mental Illness, BDD also represents Bipolar Disorder, and GAD_PD signifies Generalized Anxiety Disorder or Panic Disorder. Regarding the treatment type, BS denotes brain stimulation.

**Table 2:** Moderator Meta-Regression Analyses.

Term	*b*	*SE*	*t*	*p*	95 % CI	Model
Percentage Responders	1.02	0.00	18.46	< 0.001***	[1.02, 1.03]	Model 1
Age	1.00	0.01	−0.76	0.450	[0.98, 1.01]	Model 2
N	1.00	0.00	−0.01	0.995	[1.00, 1.00]	Model 3
Tx Duration (Weeks)	1.01	0.00	2.83	< 0.01**	[1.00, 1.02]	Model 4
Imbalance Preprocessing (Yes)	0.59	0.21	−2.52	<0.05*	[0.40, 0.89]	Model 5
Feature Type (Combined)	0.75	0.10	−3.00	< 0.01**	[0.62, 0.90]	Model 6
Feature Type (Behavior)	0.70	0.10	−3.62	< 0.001***	[0.58, 0.85]	Model 6
Feature Selection (None)	0.95	0.06	−1.00	0.318	[0.85, 1.05]	Model 7
Validation Type (CV/Nested CV)	1.37	0.10	3.10	< 0.01 **	[1.12, 1.67]	Model 8
Missing Data Solution (Yes)	0.58	0.10	−5.22	< 0.001***	[0.48, 0.71]	Model 9
Algorithm (GB)	0.86	0.18	−0.88	0.380	[0.61, 1.21]	Model 10
Algorithm (Neural Net)	0.99	0.17	−0.09	0.932	[0.71, 1.37]	Model 10
Algorithm (Other)	0.94	0.21	−0.31	0.758	[0.62, 1.41]	Model 10
Algorithm (SVM)	1.04	0.13	0.33	0.744	[0.81, 1.34]	Model 10
Algorithm (Tree)	0.80	0.14	−1.67	0.095	[0.61, 1.04]	Model 10
Ensemble Approach (Yes)	0.80	0.08	−2.83	< 0.01**	[0.69, 0.93]	Model 11
Diagnosis (Depression)	0.70	0.02	−21.54	< 0.001***	[0.67, 0.72]	Model 12
Tx Type (Medicine)	0.77	0.14	−1.88	0.060	[0.58, 1.01]	Model 13
Tx Type (Other)	0.83	0.28	−0.66	0.512	[0.48, 1.44]	Model 13
Tx Type (Psychotherapy)	0.81	0.16	−1.27	0.205	[0.59, 1.12]	Model 13
Outcome (Response)	1.07	0.05	1.27	0.202	[0.96, 1.19]	Model 14
Ethnicity (Western)	0.62	0.12	−3.96	< 0.001***	[0.49, 0.79]	Model 15
Sex (% Female)	0.997	0.00	−1.83	0.068	[0.995, 1.0002]	Model 16

*Notes*: Definitions of abbreviations are as follows: *b* = Beta Value; *SE* = Standard Error; CI = Confidence Interval;Tx = Treatment; CV = Cross Validation; GB = Gradient Boosting; SVM = Support Vector Machine. All coefficients are exponentiated values to represent odds ratios.

Reference variables for models with categorical variables: Model 5 = no; model 6 = biological; model 7 = yes; model 8 = simple Split; model 9 = no; model 10 = regression-based algorithms; model 11 = no; model 12 = non-depression disorders (e.g., anxiety); model 13 = bio-stimulation; model 14 = remission; model 15 = non-Western

Model 3 included both a random intercept and coefficient term, as the number of subjects varied across models presented within the same studies. Models 1 and 5 included sample size (N) as a covariate to control for its confounding influence, though for ease of presentation those coefficients are omitted from the table.

* ≤0.05.

** ≤0.01.

*** ≤0.001.

## Appendix A. Supplementary data

Supplementary data to this article can be found online at https://doi.org/10.1016/j.cpr.2025.102593.

## References

[R1] Abd Elrahman SM, & Abraham A (2013). A review of class imbalance problem. Journal of Network and Innovative Computing, 1, 332–340.

[R2] Aderka IM, Kauffmann A, Shalom JG, Beard C, & Björgvinsson T (2021). Using machine-learning to predict sudden gains in treatment for major depressive disorder. Behaviour Research and Therapy, 144, Article 103929. 34233251 10.1016/j.brat.2021.103929

[R3] Arends L, Hamza T, Van Houwelingen J, Heijenbrok-Kal M, Hunink M, & Stijnen T (2008). Bivariate random effects meta-analysis of ROC curves. Medical Decision Making, 28, 621–638. 18591542 10.1177/0272989X08319957

[R4] Athreya A, Iyer R, Neavin D, Wang L, Weinshilboum R, Kaddurah-Daouk R, … Bobo W (2018). Augmentation of physician assessments with multi-omics enhances predictability of drug response: A case study of major depressive disorder. IEEE Computational Intelligence Magazine, 13(3), 20–31. 30467458 10.1109/MCI.2018.2840660 PMC6241311

[R5] Athreya AP, Neavin D, Carrillo-Roa T, Skime M, Biernacka J, Frye MA, … Bobo WV (2019). Pharmacogenomics-driven prediction of antidepressant treatment outcomes: A machine-learning approach with multi-trial replication. Clinical Pharmacology & Therapeutics, 106(4), 855–865. 31012492 10.1002/cpt.1482 PMC6739122

[R6] Bailey NW, Hoy KE, Rogasch NC, Thomson RH, McQueen S, Elliot D, … Fitzgerald PB (2018). Responders to rTMS for depression show increased fronto midline theta and theta connectivity compared to non-responders. Brain Stimulation, 11(1), 190–203. 29128490 10.1016/j.brs.2017.10.015

[R7] Bailey NW, Hoy KE, Rogasch NC, Thomson RH, McQueen S, Elliot D, … Fitzgerald PB (2019). Differentiating responders and non-responders to rTMS treatment for depression after one week using resting EEG connectivity measures. Journal of Affective Disorders, 242, 68–79. 30172227 10.1016/j.jad.2018.08.058

[R8] Ball TM, Stein MB, Ramsawh HJ, Campbell-Sills L, & Paulus MP (2014). Single-subject anxiety treatment outcome prediction using functional neuroimaging. Neuropsychopharmacology, 39(5), 1254–1261. 24270731 10.1038/npp.2013.328 PMC3957121

[R9] Bao Z, Zhao X, Li J, Zhang G, Wu H, Ning Y, … Yang Z (2021). Prediction of repeated-dose intravenous ketamine response in major depressive disorder using the GWAS-based machine learning approach. Journal of Psychiatric Research, 138, 284–290. 33878621 10.1016/j.jpsychires.2021.04.014

[R10] Bartlett EA, DeLorenzo C, Sharma P, Yang J, Zhang M, Petkova E, … Parsey RV (2018). Pretreatment and early-treatment cortical thickness is associated with SSRI treatment response in major depressive disorder. Neuropsychopharmacology, 43(11), 2221–2230. 29955151 10.1038/s41386-018-0122-9 PMC6135779

[R11] Bartova L, Dold M, Kautzky A, Fabbri C, Spies M, Serretti A, … Kasper S (2019). Results of the European group for the study of resistant depression (GSRD)—Basis for further research and clinical practice. The World Journal of Biological Psychiatry, 20(6), 427–448. 31340696 10.1080/15622975.2019.1635270

[R12] Begg CB, & Mazumdar M (1994). Operating characteristics of a rank correlation test for publication bias. Biometrics, 50(4), 1088–1101. 7786990

[R13] Benedetti F, Poletti S, Vai B, Mazza MG, Lorenzi C, Brioschi S, … Zanardi R (2021). Higher baseline interleukin-1β and TNF-α hamper antidepressant response in major depressive disorder. European Neuropsychopharmacology, 42, 35–44. 33191075 10.1016/j.euroneuro.2020.11.009

[R14] Benoit JR, Dursun SM, Greiner R, Cao B, Brown MR, Lam RW, & Greenshaw AJ (2022). Using machine learning to predict remission in patients with major depressive disorder treated with desvenlafaxine. The Canadian Journal of Psychiatry, 67(1), 39–47. 34379019 10.1177/07067437211037141 PMC8808003

[R15] Bertie LA, Quiroz JC, Berkovsky S, Arendt K, Bögels S, Coleman JR, … Hudson JL (2024). Predicting remission following CBT for childhood anxiety disorders: A machine learning approach (pp. 1–11). Psychological Medicine. 10.1017/S0033291724002654 39686883

[R16] Bi Y, Ren D, Guo Z, Ma G, Xu F, Chen Z, … He G (2021). Influence and interaction of genetic, cognitive, neuroendocrine and personalistic markers to antidepressant response in Chinese patients with major depression. Progress in Neuro Psychopharmacology and Biological Psychiatry, 104, Article 110036. 32702381 10.1016/j.pnpbp.2020.110036

[R17] Bian A, Xiao F, Kong X, Ji X, Fang S, He J, … ZIB Consortium. (2024). Predictive modeling of antidepressant efficacy based on cognitive neuropsychological theory. Journal of Affective Disorders, 354, 563–573. 38484886 10.1016/j.jad.2024.03.029

[R18] Bone C, Simmonds-Buckley M, Thwaites R, Sandford D, Merzhvynska M, Rubel J, … Delgadillo J (2021). Dynamic prediction of psychological treatment outcomes: Development and validation of a prediction model using routinely collected symptom data. The Lancet Digital Health, 3(4), e231–e240. 33766287 10.1016/S2589-7500(21)00018-2

[R19] Bossarte RM, Ross EL, Liu H, Turner B, Bryant C, Zainal NH, … Kessler RC (2023). Development of a model to predict combined antidepressant medication and psychotherapy treatment response for depression among veterans. Journal of Affective Disorders, 326, 111–119. 36709831 10.1016/j.jad.2023.01.082 PMC9975041

[R20] Brown EC, Clark DL, Forkert ND, Molnar CP, Kiss ZH, & Ramasubbu R (2020). Metabolic activity in subcallosal cingulate predicts response to deep brain stimulation for depression. Neuropsychopharmacology, 45(10), 1681–1688. 32580207 10.1038/s41386-020-0745-5 PMC7419290

[R21] Browning M, Kingslake J, Dourish CT, Goodwin GM, Harmer CJ, & Dawson GR (2019). Predicting treatment response to antidepressant medication using early changes in emotional processing. European Neuropsychopharmacology, 29(1), 66–75. 30473402 10.1016/j.euroneuro.2018.11.1102

[R22] Bruin WB, Oltedal L, Bartsch H, Abbott C, Argyelan M, Barbour T, … Van Wingen G (2024). Development and validation of a multimodal neuroimaging biomarker for electroconvulsive therapy outcome in depression: A multicenter machine learning analysis. Psychological Medicine, 54(3), 495–506. 37485692 10.1017/S0033291723002040

[R23] Calabrò M, Fabbri C, Serretti A, Kasper S, Zohar J, Souery D, Crisafulli C (2025). A machine learning approach to predict treatment efficacy and adverse effects in major depression using CYP2C19 and clinical-environmental predictors. Psychiatric Genetics, 35(2), 17–25. 40008580 10.1097/YPG.0000000000000379

[R24] Cao B, Luo Q, Fu Y, Du L, Qiu T, Yang X, … Qiu H (2018). Predicting individual responses to the electroconvulsive therapy with hippocampal subfield volumes in major depression disorder. Scientific Reports, 8(1), 5434. 29615675 10.1038/s41598-018-23685-9 PMC5882798

[R25] Carr E, Rietschel M, Mors O, Henigsberg N, Aitchison KJ, Maier W, … Iniesta R (2025). Optimizing the prediction of depression remission: A longitudinal machine learning approach. American Journal of Medical Genetics Part B: Neuropsychiatric Genetics, 198(3), Article e33014. 10.1002/ajmg.b.33014 39470297

[R26] Carrillo F, Sigman M, Slezak DF, Ashton P, Fitzgerald L, Stroud J, … Carhart-Harris RL (2018). Natural speech algorithm applied to baseline interview data can predict which patients will respond to psilocybin for treatment-resistant depression. Journal of Affective Disorders, 230, 84–86. 29407543 10.1016/j.jad.2018.01.006

[R27] Cearns M, Amare AT, Schubert KO, Thalamuthu A, Frank J, Streit F, … Baune BT (2022). Using polygenic scores and clinical data for bipolar disorder patient stratification and lithium response prediction: Machine learning approach. The British Journal of Psychiatry, 220(4), 219–228. 35225756 10.1192/bjp.2022.28

[R28] Chai KE, Graham-Schmidt K, Lee CM, Rock D, Coleman M, Betts KS, … McEvoy PM (2024). Predicting anxiety treatment outcome in community mental health services using linked health administrative data. Scientific Reports, 14(1), 20559. 39232215 10.1038/s41598-024-71557-2 PMC11375212

[R29] Chang B, Choi Y, Jeon M, Lee J, Han KM, Kim A, … Kang J (2019). ARPNet: Antidepressant response prediction network for major depressive disorder. Genes, 10(11), 907. 31703457 10.3390/genes10110907 PMC6895829

[R30] Chekroud AM, Bondar J, Delgadillo J, Doherty G, Wasil A, Fokkema M, … Choi K (2021). The promise of machine learning in predicting treatment outcomes in psychiatry. World Psychiatry, 20(2), 154–170. 34002503 10.1002/wps.20882 PMC8129866

[R31] Chekroud AM, Zotti RJ, Shehzad Z, Gueorguieva R, Johnson MK, Trivedi MH, … Corlett PR (2016). Cross-trial prediction of treatment outcome in depression: A machine learning approach. The Lancet Psychiatry, 3(3), 243–250. 26803397 10.1016/S2215-0366(15)00471-X

[R32] Chen B, Jiao Z, Shen T, Fan R, Chen Y, & Xu Z (2023). Early antidepressant treatment response prediction in major depression using clinical and TPH2 DNA methylation features based on machine learning approaches. BMC Psychiatry, 23(1), 299. 37127594 10.1186/s12888-023-04791-z PMC10150459

[R33] Cheung MW (2014). Modeling dependent effect sizes with three-level meta-analyses: A structural equation modeling approach. Psychological Methods, 19(2), 211–229. 23834422 10.1037/a0032968

[R34] Choi KM, Lee T, Im CH, & Lee SH (2024). Prediction of pharmacological treatment efficacy using electroencephalography-based salience network in patients with major depressive disorder. Frontiers in Psychiatry, 15, 1469645. 39483735 10.3389/fpsyt.2024.1469645 PMC11525785

[R35] Christ NM, Schubert RA, Mundle R, Pridgen S, & Held P (2023). Using machine learning to predict sudden gains in intensive treatment for PTSD. Journal of Anxiety Disorders, 100, Article 102783. 37871453 10.1016/j.janxdis.2023.102783

[R36] Coley RY, Boggs JM, Beck A, & Simon GE (2021). Predicting outcomes of psychotherapy for depression with electronic health record data. Journal of Affective Disorders Reports, 6, Article 100198. 34541567 10.1016/j.jadr.2021.100198 PMC8448296

[R37] Copa D, Erritzoe D, Giribaldi B, Nutt D, Carhart-Harris R, & Tagliazucchi E (2024). Predicting the outcome of psilocybin treatment for depression from baseline fMRI functional connectivity. Journal of Affective Disorders, 353, 60–69. 38423367 10.1016/j.jad.2024.02.089

[R38] Corlier J, Wilson A, Hunter AM, Vince-Cruz N, Krantz D, Levitt J, … Leuchter AF (2019). Changes in functional connectivity predict outcome of repetitive transcranial magnetic stimulation treatment of major depressive disorder. Cerebral Cortex, 29(12), 4958–4967. 30953441 10.1093/cercor/bhz035 PMC7305800

[R39] Curtiss J, Smoller JW, & Pedrelli P (2024). Optimizing precision medicine for second-step depression treatment: A machine learning approach. Psychological Medicine, 1–8. 10.1017/S0033291724000497 38533794

[R40] Curtiss JE, Bernstein EE, Wilhelm S, & Phillips KA (2023). Predictors of pharmacotherapy outcomes for body dysmorphic disorder: A machine learning approach. Psychological Medicine, 53(8), 3366–3376. 35000652 10.1017/S0033291721005390 PMC9836197

[R41] Delgadillo J, Duhne GS, & P.. (2020). Targeted prescription of cognitive–behavioral therapy versus person-centered counseling for depression using a machine learning approach. Journal of Consulting and Clinical Psychology, 88(1), 14. 31841021 10.1037/ccp0000476

[R42] Díaz-Zuluaga AM, Vélez JI, Cuartas M, Valencia J, Castaño M, Palacio JD, López-Jaramillo C (2023). Ancestry component as a major predictor of lithium response in the treatment of bipolar disorder. Journal of Affective Disorders, 332, 203–209. 36997125 10.1016/j.jad.2023.03.058

[R43] Doebler P. (2015). Meta-analysis of diagnostic accuracy (version 0.5.11). Software. Retrieved from http://cran.r-project.org/web/packages/mada/mada.pdf.

[R44] Doehrmann O, Ghosh SS, Polli FE, Reynolds GO, Horn F, Keshavan A, … Gabrieli JD (2013). Predicting treatment response in social anxiety disorder from functional magnetic resonance imaging. JAMA Psychiatry, 70(1), 87–97. 22945462 10.1001/2013.jamapsychiatry.5 PMC3844518

[R45] van der Does Y, Turner RJ, Bartels MJ, Hagoort K, Metselaar A, Scheepers F, … van Dellen E (2023). Outcome prediction of electroconvulsive therapy for depression. Psychiatry Research, 326, Article 115328. 37429173 10.1016/j.psychres.2023.115328

[R46] Dougherty RF, Clarke P, Atli M, Kuc J, Schlosser D, Dunlop BW, … Ryslik GA (2023). Psilocybin therapy for treatment resistant depression: Prediction of clinical outcome by natural language processing. Psychopharmacology, 1–9. 10.1007/s00213-023-06432-5 PMC12226623 37606733

[R47] Drysdale AT, Grosenick L, Downar J, Dunlop K, Mansouri F, Meng Y, … Liston C (2017). Resting-state connectivity biomarkers define neurophysiological subtypes of depression. Nature Medicine, 23(1), 28–38. 10.1038/nm.4246 PMC5624035 27918562

[R48] Duan J, Li Y, Zhang X, Dong S, Zhao P, Liu J, … Wang F (2023). Predicting treatment response in adolescents and young adults with major depressive episodes from fMRI using graph isomorphism network. NeuroImage: Clinical, 40, Article 103534. 37939442 10.1016/j.nicl.2023.103534 PMC10665904

[R49] Ebrahimzadeh E, Dehghani A, Asgarinejad M, & Soltanian-Zadeh H (2024). Non-linear processing and reinforcement learning to predict rTMS treatment response in depression. Psychiatry Research: Neuroimaging, 337, Article 111764. 38043370 10.1016/j.pscychresns.2023.111764

[R50] Ebrahimzadeh E, Fayaz F, Rajabion L, Seraji M, Aflaki F, Hammoud A, … Soltanian Zadeh H (2023). Machine learning approaches and non-linear processing of extracted components in frontal region to predict rTMS treatment response in major depressive disorder. Frontiers in Systems Neuroscience, 17, Article 919977. 36968455 10.3389/fnsys.2023.919977 PMC10034109

[R51] Flygare O, Enander J, Andersson E, Ljótsson B, Ivanov VZ, Mataix-Cols D, & Rück C (2020). Predictors of remission from body dysmorphic disorder after internet-delivered cognitive behavior therapy: A machine learning approach. BMC Psychiatry, 20, 1–9. 32429939 10.1186/s12888-020-02655-4 PMC7238519

[R52] Forrest LN, Ivezaj V, & Grilo CM (2023). Machine learning v. traditional regression models predicting treatment outcomes for binge-eating disorder from a randomized controlled trial. Psychological Medicine, 53(7), 2777–2788. 34819195 10.1017/S0033291721004748 PMC9130342

[R53] Gabrieli JD, Ghosh SS, & Whitfield-Gabrieli S (2015). Prediction as a humanitarian and pragmatic contribution from human cognitive neuroscience. Neuron, 85(1), 11–26. 25569345 10.1016/j.neuron.2014.10.047 PMC4287988

[R54] Gao C, Xu Z, Tan T, Chen Z, Shen T, Chen L, … Yuan Y (2022). Combination of spontaneous regional brain activity and HTR1A/1B DNA methylation to predict early responses to antidepressant treatments in MDD. Journal of Affective Disorders, 302(249), 257. 10.1016/j.jad.2022.01.098 35092755

[R55] Gärtner M, Ghisu E, Herrera-Melendez AL, Koslowski M, Aust S, Asbach P, … Bajbouj M (2021). Using routine MRI data of depressed patients to predict individual responses to electroconvulsive therapy. Experimental Neurology, 335, Article 113505. 33068570 10.1016/j.expneurol.2020.113505

[R56] Gillett G, Tomlinson A, Efthimiou O, & Cipriani A (2020). Predicting treatment effects in unipolar depression: A meta-review. Pharmacology & Therapeutics, 212, Article 107557. 32437828 10.1016/j.pharmthera.2020.107557

[R57] Goyal RK, Kalaria SN, McElroy SL, & Gopalakrishnan M (2022). An exploratory machine learning approach to identify placebo responders in pharmacological binge eating disorder trials. Clinical and Translational Science, 15(12), 2878–2887. 36126231 10.1111/cts.13406 PMC9747128

[R58] Grassi M, Rickelt J, Caldirola D, Eikelenboom M, van Oppen P, Dumontier M, … Schruers K (2022). Prediction of illness remission in patients with obsessive compulsive disorder with supervised machine learning. Journal of Affective Disorders, 296, 117–125. 34600172 10.1016/j.jad.2021.09.042

[R59] Grzenda A, Speier W, Siddarth P, Pant A, Krause-Sorio B, Narr K, & Lavretsky H (2021). Machine learning prediction of treatment outcome in late-life depression. Frontiers in Psychiatry, 12, Article 738494. 34744829 10.3389/fpsyt.2021.738494 PMC8563624

[R60] Guilloux JP, Bassi S, Ding Y, Walsh C, Turecki G, Tseng G, … Sibille E (2015). Testing the predictive value of peripheral gene expression for nonremission following citalopram treatment for major depression. Neuropsychopharmacology, 40(3), 701–710. 25176167 10.1038/npp.2014.226 PMC4289958

[R61] Hahn T, Kircher T, Straube B, Wittchen HU, Konrad C, Ströhle A, … Lueken U (2015). Predicting treatment response to cognitive behavioral therapy in panic disorder with agoraphobia by integrating local neural information. JAMA Psychiatry, 72(1), 68–74. 25409415 10.1001/jamapsychiatry.2014.1741

[R62] Hammelrath L, Hilbert K, Heinrich M, Zagorscak P, & Knaevelsrud C (2024). Select or adjust? How information from early treatment stages boosts the prediction of non response in internet-based depression treatment. Psychological Medicine, 54(8), 1641 1650. 38087867 10.1017/S0033291723003537

[R63] Harrer M, Cuijpers P, Furukawa TA, & Ebert DD (2021). Doing meta-analysis with R: A hands-on guide. Boca Raton, FL and London: Chapman & Hall/CRC Press. ISBN 978-0-367-61007-4.

[R64] Harris JK, Hassel S, Davis AD, Zamyadi M, Arnott SR, Milev R, … Greiner R (2022). Predicting escitalopram treatment response from pre-treatment and early response resting state fMRI in a multi-site sample: A CAN-BIND-1 report. NeuroImage: Clinical, 35, Article 103120. 35908308 10.1016/j.nicl.2022.103120 PMC9421454

[R65] Hasanzadeh F, Mohebbi M, & Rostami R (2019). Prediction of rTMS treatment response in major depressive disorder using machine learning techniques and nonlinear features of EEG signal. Journal of Affective Disorders, 256, 132–142. 31176185 10.1016/j.jad.2019.05.070

[R66] Held P, Schubert RA, Pridgen S, Kovacevic M, Montes M, Christ NM, … Smith DL (2022). Who will respond to intensive PTSD treatment? A machine learning approach to predicting response prior to starting treatment. Journal of Psychiatric Research, 151, 78–85. 35468429 10.1016/j.jpsychires.2022.03.066

[R67] Hilbert K, Böhnlein J, Meinke C, Chavanne AV, Langhammer T, Stumpe L, Lueken U (2024). Lack of evidence for predictive utility from resting state fMRI data for individual exposure-based cognitive behavioral therapy outcomes: A machine learning study in two large multi-site samples in anxiety disorders. NeuroImage, 295, Article 120639. 38796977 10.1016/j.neuroimage.2024.120639

[R68] Hilbert K, Jacobi T, Kunas SL, Elsner B, Reuter B, Lueken U, & Kathmann N (2021). Identifying CBT non-response among OCD outpatients: A machine-learning approach. Psychotherapy Research, 31(1), 52–62. 33175642 10.1080/10503307.2020.1839140

[R69] Ho CSH, Wang J, Tay GWN, Ho R, Lin H, Li Z, & Chen N (2025). Application of functional near-infrared spectroscopy and machine learning to predict treatment response after six months in major depressive disorder. Translational Psychiatry, 15(1), 7. 39799114 10.1038/s41398-025-03224-7 PMC11724951

[R70] Hofmann SG, & Curtiss J (2018). A complex network approach to clinical science. European Journal of Clinical Investigation, 48(8), Article e12986. 29931701 10.1111/eci.12986

[R71] Hofmann SG, Curtiss JE, & Hayes SC (2020). Beyond linear mediation: Toward a dynamic network approach to study treatment processes. Clinical Psychology Review, 76, Article 101824. 32035297 10.1016/j.cpr.2020.101824 PMC7137783

[R72] Hoogendoorn M, Berger T, Schulz A, Stolz T, & Szolovits P (2016). Predicting social anxiety treatment outcome based on therapeutic email conversations. IEEE Journal of Biomedical and Health Informatics, 21(5), 1449–1459. 27542187 10.1109/JBHI.2016.2601123 PMC5613669

[R73] Hopman HJ, Chan SMS, Chu WCW, Lu H, Tse CY, Chau SWH, … Neggers SFW (2021). Personalized prediction of transcranial magnetic stimulation clinical response in patients with treatment-refractory depression using neuroimaging biomarkers and machine learning. Journal of Affective Disorders, 290, 261–271. 34010751 10.1016/j.jad.2021.04.081

[R74] Hornstein S, Forman-Hoffman V, Nazander A, Ranta K, & Hilbert K (2021). Predicting therapy outcome in a digital mental health intervention for depression and anxiety: A machine learning approach. DIGITAL HEALTH, 7, 20552076211060659. 10.1177/20552076211060659 PMC8637697 34868624

[R75] Hosmer DW, & Lemeshow S (2000). Assessing fit of the model. In Hosmer D, & Lemeshow S (Eds.), Applied logistic regression (pp. 143–160). New York (NY): Wiley.

[R76] Iniesta R, Malki K, Maier W, Rietschel M, Mors O, Hauser J, … Uher R (2016). Combining clinical variables to optimize prediction of antidepressant treatment outcomes. Journal of Psychiatric Research, 78, 94–102. 27089522 10.1016/j.jpsychires.2016.03.016

[R77] Jaworska N, De la Salle S, Ibrahim MH, Blier P, & Knott V (2019). Leveraging machine learning approaches for predicting antidepressant treatment response using electroencephalography (EEG) and clinical data. Frontiers in Psychiatry, 9, 768. 30692945 10.3389/fpsyt.2018.00768 PMC6339954

[R78] Joyce JB, Grant CW, Liu D, MahmoudianDehkordi S, Kaddurah-Daouk R, Skime M, … Athreya AP (2021). Multi-omics driven predictions of response to acute phase combination antidepressant therapy: A machine learning approach with cross-trial replication. Translational Psychiatry, 11(1), 513. 34620827 10.1038/s41398-021-01632-z PMC8497535

[R79] Ju Y, Wang M, Liu J, Liu B, Yan D, Lu X, … Li L (2023). Modulation of resting-state functional connectivity in default mode network is associated with the long-term treatment outcome in major depressive disorder. Psychological Medicine, 53(13), 5963 5975. 36164996 10.1017/S0033291722002628

[R80] Kambeitz J, Goerigk S, Gattaz W, Falkai P, Bensenor IM, Lotufo PA, … Brunoni AR (2020). Clinical patterns differentially predict response to transcranial direct current stimulation (tDCS) and escitalopram in major depression: A machine learning analysis of the ELECT-TDCS study. Journal of Affective Disorders, 265, 460–467. 32090773 10.1016/j.jad.2020.01.118

[R81] Kannampallil T, Dai R, Lv N, Xiao L, Lu C, Ajilore OA, … Ma J (2022). Cross-trial prediction of depression remission using problem-solving therapy: A machine learning approach. Journal of Affective Disorders, 308, 89–97. 35398399 10.1016/j.jad.2022.04.015

[R82] Kautzky A, Dold M, Bartova L, Spies M, Vanicek T, Souery D, … Kasper S (2017). Refining prediction in treatment-resistant depression: Results of machine learning analyses in the TRD III sample. The Journal of Clinical Psychiatry, 79(1), 14989. 10.4088/JCP.16m11385 29228516

[R83] Kautzky A, Möller HJ, Dold M, Bartova L, Seemüller F, Laux G, Kasper S (2021). Combining machine learning algorithms for prediction of antidepressant treatment response. Acta Psychiatrica Scandinavica, 143(1), 36–49. 33141944 10.1111/acps.13250 PMC7839691

[R84] Khodayari-Rostamabad A, Reilly JP, Hasey GM, de Bruin H, & MacCrimmon DJ (2013). A machine learning approach using EEG data to predict response to SSRI treatment for major depressive disorder. Clinical Neurophysiology, 124(10), 1975–1985. 23684127 10.1016/j.clinph.2013.04.010

[R85] Kim JS, Wang B, Kim M, Lee J, Kim H, Roh D, … Ryan N (2023). Prediction of diagnosis and treatment response in adolescents with depression by using a smartphone app and deep learning approaches: Usability study. JMIR Formative Research, 7(1), Article e45991. 37223978 10.2196/45991 PMC10248781

[R86] Kim S, Kang Y, Shin H, Lee EB, Ham BJ, & Choi Y (2024). Liquid biopsy-based detection and response prediction for depression. ACS Nano, 18(47), 32498–32507. 39501510 10.1021/acsnano.4c08233 PMC11604100

[R87] Kim S, Yang C, Dong SY, & Lee SH (2022). Predictions of tDCS treatment response in PTSD patients using EEG based classification. Frontiers in Psychiatry, 13, Article 876036. 35845448 10.3389/fpsyt.2022.876036 PMC9277561

[R88] Kong Y, Gao S, Yue Y, Hou Z, Shu H, Xie C, … Yuan Y (2021). Spatio-temporal graph convolutional network for diagnosis and treatment response prediction of major depressive disorder from functional connectivity. Human Brain Mapping, 42(12), 3922 3933. 33969930 10.1002/hbm.25529 PMC8288094

[R89] Kuhn M, & Johnson K (2013). Applied predictive modeling. Springer Science & Business Media.

[R90] Kuhn M, & Johnson K (2019). Feature engineering and selection: A practical approach for predictive models. New York: CRC Press.

[R91] Leaver AM, Wade B, Vasavada M, Hellemann G, Joshi SH, Espinoza R, & Narr KL (2018). Fronto-temporal connectivity predicts ECT outcome in major depression. Frontiers in Psychiatry, 9, 92. 29618992 10.3389/fpsyt.2018.00092 PMC5871748

[R92] Lee DY, Kim N, Park C, Gan S, Son SJ, Park RW, & Park B (2024a). Explainable multimodal prediction of treatment-resistance in patients with depression leveraging brain morphometry and natural language processing. Psychiatry Research, 334, Article 115817. 38430816 10.1016/j.psychres.2024.115817

[R93] Lee LH, Ho CSH, Chan YL, Tay GWN, Lu CK, & Tang TB (2024b). Antidepressant treatment response prediction with early assessment of functional near-infrared spectroscopy and micro-RNA. IEEE Journal of Translational Engineering in Health and Medicine., 13, 9–22. 39911775 10.1109/JTEHM.2024.3506556 PMC11793863

[R94] Lee Y, Ragguett RM, Mansur RB, Boutilier JJ, Rosenblat JD, Trevizol A, … McIntyre RS (2018). Applications of machine learning algorithms to predict therapeutic outcomes in depression: A meta-analysis and systematic review. Journal of Affective Disorders, 241, 519–532. 30153635 10.1016/j.jad.2018.08.073

[R95] Leehr EJ, Roesmann K, Böhnlein J, Dannlowski U, Gathmann B, Herrmann MJ, … Hilbert K (2021). Clinical predictors of treatment response towards exposure therapy in virtuo in spider phobia: A machine learning and external cross-validation approach. Journal of Anxiety Disorders, 83, Article 102448. 34298236 10.1016/j.janxdis.2021.102448

[R96] Lemmens LH, Van Bronswijk SC, Peeters F, Arntz A, Hollon SD, & Huibers MJ (2019). Long-term outcomes of acute treatment with cognitive therapy v. interpersonal psychotherapy for adult depression: Follow-up of a randomized controlled trial. Psychological Medicine, 49(3), 465–473. 29792234 10.1017/S0033291718001083

[R97] Lenhard F, Sauer S, Andersson E, Månsson KN, Mataix-Cols D, Rück C, & Serlachius E (2018). Prediction of outcome in internet-delivered cognitive behaviour therapy for paediatric obsessive-compulsive disorder: A machine learning approach. International Journal of Methods in Psychiatric Research, 27(1), Article e1576. 28752937 10.1002/mpr.1576 PMC6877165

[R98] Li X, Guo J, Chen X, Yu R, Chen W, Zheng A, … Kuang L (2023a). Predicting responses to electroconvulsive therapy in adolescents with treatment-refractory depression based on resting-state fMRI. Journal of Clinical Medicine, 12(10), 3556. 37240663 10.3390/jcm12103556 PMC10218878

[R99] Li CT, Chen CS, Cheng CM, Chen CP, Chen JP, Chen MH, … Tsai SJ (2023b). Prediction of antidepressant responses to non-invasive brain stimulation using frontal electroencephalogram signals: Cross-dataset comparisons and validation. Journal of Affective Disorders, 343, 86–95. 37579885 10.1016/j.jad.2023.08.059

[R100] Lin E, Kuo PH, Liu YL, Yu YWY, Yang AC, & Tsai SJ (2018). A deep learning approach for predicting antidepressant response in major depression using clinical and genetic biomarkers. Frontiers in Psychiatry, 9, 290. 30034349 10.3389/fpsyt.2018.00290 PMC6043864

[R101] Lin E, Kuo PH, Liu YL, Yu YWY, Yang AC, & Tsai SJ (2020). Prediction of antidepressant treatment response and remission using an ensemble machine learning framework. Pharmaceuticals, 13(10), 305. 33065962 10.3390/ph13100305 PMC7599952

[R102] Lissemore JI, Mulsant BH, Bonner AJ, Butters MA, Chen R, Downar J, … Blumberger DM (2022). Transcranial magnetic stimulation indices of cortical excitability enhance the prediction of response to pharmacotherapy in late-life depression. Biological Psychiatry: Cognitive Neuroscience and Neuroimaging, 7(3), 265 275. 34311121 10.1016/j.bpsc.2021.07.005 PMC8783923

[R103] Loerinc AG, Meuret AE, Twohig MP, Rosenfield D, Bluett EJ, & Craske MG (2015). Response rates for CBT for anxiety disorders: Need for standardized criteria. Clinical Psychology Review, 42, 72–82. 26319194 10.1016/j.cpr.2015.08.004

[R104] LoParo D, Dunlop BW, Nemeroff CB, Mayberg HS, & Craighead WE (2025). Prediction of individual patient outcomes to psychotherapy vs medication for major depression. npj Mental Health Research, 4(1), 4. 39910171 10.1038/s44184-025-00119-9 PMC11799290

[R105] Lu Z, Wang J, Wang F, & Wu Z (2023). Application of graph frequency attention convolutional neural networks in depression treatment response. Frontiers in Psychiatry, 14, 1244208. 38045613 10.3389/fpsyt.2023.1244208 PMC10690947

[R106] Ma H, Zhang D, Wang Y, Ding Y, Yang J, & Li K (2023). Prediction of early improvement of major depressive disorder to antidepressant medication in adolescents with radiomics analysis after ComBat harmonization based on multiscale structural MRI. BMC Psychiatry, 23(1), 466. 37365541 10.1186/s12888-023-04966-8 PMC10294484

[R107] Maciukiewicz M, Marshe VS, Hauschild AC, Foster JA, Rotzinger S, Kennedy JL, … Geraci J (2018). GWAS-based machine learning approach to predict duloxetine response in major depressive disorder. Journal of Psychiatric Research, 99, 62–68. 29407288 10.1016/j.jpsychires.2017.12.009

[R108] Månsson KN, Frick A, Boraxbekk CJ, Marquand AF, Williams SCR, Carlbring P, … Furmark T (2015). Predicting long-term outcome of internet-delivered cognitive behavior therapy for social anxiety disorder using fMRI and support vector machine learning. Translational Psychiatry, 5(3), e530. 25781229 10.1038/tp.2015.22 PMC4354352

[R109] Marrero-Polanco J, Joyce JB, Grant CW, Croarkin PE, Athreya AP, & Bobo WV (2025). Predicting remission after acute phase pharmacotherapy in patients with bipolar I depression: A machine learning approach with cross-trial and cross-drug replication. Bipolar Disorders, 27(1), 36–46. 39362832 10.1111/bdi.13506 PMC11848014

[R110] Mathai DS, Hull TD, Vando L, & Malgaroli M (2024). At-home, telehealth-supported ketamine treatment for depression: Findings from longitudinal, machine learning and symptom network analysis of real-world data. Journal of Affective Disorders, 361, 198–208. 38810787 10.1016/j.jad.2024.05.131 PMC11284959

[R111] Metin SZ, Balli Altuglu T, Metin B, Erguzel TT, Yigit S, Arıkan MK, & Tarhan KN (2020). Use of EEG for predicting treatment response to transcranial magnetic stimulation in obsessive compulsive disorder. Clinical EEG and Neuroscience, 51(3), 139–145. 31583910 10.1177/1550059419879569

[R112] Metin SZ, Uyulan Ç, Farhad S, Ergüzel TT, Türk Ö, Metin B, … Tarhan N (2025). Deep learning-based artificial intelligence can differentiate treatment-resistant and responsive depression cases with high accuracy. Clinical EEG and Neuroscience, 56(2), 119–130. 39251228 10.1177/15500594241273181

[R113] Mirjebreili SM, Shalbaf R, & Shalbaf A (2024). Prediction of treatment response in major depressive disorder using a hybrid of convolutional recurrent deep neural networks and effective connectivity based on EEG signal. Physical and Engineering Sciences in Medicine, 1–10. 10.1007/s13246-024-01392-2 38358619

[R114] Mizrahi L, Choudhary A, Ofer P, Goldberg G, Milanesi E, Kelsoe JR, … Stern S (2023). Immunoglobulin genes expressed in lymphoblastoid cell lines discern and predict lithium response in bipolar disorder patients. Molecular Psychiatry, 28(10), 4280–4293. 37488168 10.1038/s41380-023-02183-z PMC10827667

[R115] Mumtaz W, Xia L, Mohd Yasin MA, Azhar Ali SS, & Malik AS (2017). A wavelet based technique to predict treatment outcome for major depressive disorder. PLoS One, 12(2), Article e0171409. 28152063 10.1371/journal.pone.0171409 PMC5289714

[R116] Nakajima K, Takamiya A, Uchida T, Kudo S, Nishida H, Minami F, … Hirano J (2022). Individual prediction of remission based on clinical features following electroconvulsive therapy: A machine learning approach. The Journal of Clinical Psychiatry, 83(5), 42434. 10.4088/JCP.21m14293 36005893

[R117] Nguyen KP, Fatt CC, Treacher A, Mellema C, Cooper C, Jha MK, … Montillo AA (2022). Patterns of pretreatment reward task brain activation predict individual antidepressant response: Key results from the EMBARC randomized clinical trial. Biological Psychiatry, 91(6), 550–560. 34916068 10.1016/j.biopsych.2021.09.011 PMC8857018

[R118] Nie Z, Vairavan S, Narayan VA, Ye J, & Li QS (2018). Predictive modeling of treatment resistant depression using data from STAR* D and an independent clinical study. PLoS One, 13(6), Article e0197268. 29879133 10.1371/journal.pone.0197268 PMC5991746

[R119] Nobakhsh B, Shalbaf A, Rostami R, Kazemi R, Rezaei E, & Shalbaf R (2023). An effective brain connectivity technique to predict repetitive transcranial magnetic stimulation outcome for major depressive disorder patients using EEG signals. Physical and Engineering Sciences in Medicine, 46(1), 67–81. 36445618 10.1007/s13246-022-01198-0

[R120] Nunez JJ, Nguyen TT, Zhou Y, Cao B, Ng RT, Chen J, … Lam RW (2021). Replication of machine learning methods to predict treatment outcome with antidepressant medications in patients with major depressive disorder from STAR* D and CAN-BIND-1. PLoS One, 16(6), Article e0253023. 34181661 10.1371/journal.pone.0253023 PMC8238228

[R121] Oakley T, Coskuner J, Cadwallader A, Ravan M, & Hasey G (2022). EEG biomarkers to predict response to sertraline and placebo treatment in major depressive disorder. IEEE Transactions on Biomedical Engineering, 70(3), 909–919. 10.1109/TBME.2022.3204861 36094967

[R122] Pae C, Kim HJ, Bang M, Park CI, & Lee SH (2024). Predicting treatment outcomes in patients with panic disorder: Cross-sectional and two-year longitudinal structural connectome analysis using machine learning methods. Journal of Anxiety Disorders, 106, Article 102895. 39121510 10.1016/j.janxdis.2024.102895

[R123] Patel MJ, Andreescu C, Price JC, Edelman KL, Reynolds CF III, & Aizenstein HJ (2015). Machine learning approaches for integrating clinical and imaging features in late life depression classification and response prediction. International Journal of Geriatric Psychiatry, 30(10), 1056–1067. 25689482 10.1002/gps.4262 PMC4683603

[R124] Pei C, Sun Y, Zhu J, Wang X, Zhang Y, Zhang S, … Lu Q (2020). Ensemble learning for early-response prediction of antidepressant treatment in major depressive disorder. Journal of Magnetic Resonance Imaging, 52(1), 161–171. 31859419 10.1002/jmri.27029

[R125] Perlis RH (2013). A clinical risk stratification tool for predicting treatment resistance in major depressive disorder. Biological Psychiatry, 74(1), 7–14. 23380715 10.1016/j.biopsych.2012.12.007 PMC3690142

[R126] Perlman K, Mehltretter J, Benrimoh D, Armstrong C, Fratila R, Popescu C, … Turecki G (2024). Development of a differential treatment selection model for depression on consolidated and transformed clinical trial datasets. Translational Psychiatry, 14(1), 263. 38906883 10.1038/s41398-024-02970-4 PMC11192904

[R127] Pettorruso M, Guidotti R, d’Andrea G, De Risio L, D’Andrea A, Chiappini S, … & REAL ESK Study Group. (2023). Predicting outcome with intranasal esketamine treatment: A machine-learning, three-month study in treatment-resistant depression (ESK LEARNING). Psychiatry Research, 327, Article 115378. 37574600 10.1016/j.psychres.2023.115378

[R128] Pigoni A, Delvecchio G, Turtulici N, Madonna D, Pietrini P, Cecchetti L, & Brambilla P (2024). Machine learning and the prediction of suicide in psychiatric populations: A systematic review. Translational Psychiatry, 14(1), 140. 38461283 10.1038/s41398-024-02852-9 PMC10925059

[R129] Poirot MG, Boucherie DE, Caan MW, Goya-Maldonado R, Belov V, Corruble E, … Schrantee A (2025). Predicting antidepressant treatment response from cortical structure on MRI: A mega-analysis from the ENIGMA-MDD working group. Human Brain Mapping, 46(1), Article e70053. 39757979 10.1002/hbm.70053 PMC11702469

[R130] Poirot MG, Ruhe HG, Mutsaerts HJM, Maximov II, Groote IR, Bjørnerud A, … Caan MW (2024). Treatment response prediction in major depressive disorder using multimodal MRI and clinical data: Secondary analysis of a randomized clinical trial. American Journal of Psychiatry, 181(3), 223–233. 38321916 10.1176/appi.ajp.20230206

[R131] Prasad N, Chien I, Regan T, Enrique A, Palacios J, Keegan D, … Thieme A (2023). Deep learning for the prediction of clinical outcomes in internet-delivered CBT for depression and anxiety. PLoS One, 18(11), Article e0272685. 38011176 10.1371/journal.pone.0272685 PMC10681250

[R132] Puac-Polanco V, Ziobrowski HN, Ross EL, Liu H, Turner B, Cui R, … Kessler RC (2023). Development of a model to predict antidepressant treatment response for depression among veterans. Psychological Medicine, 53(11), 5001–5011. 37650342 10.1017/S0033291722001982 PMC10519376

[R133] Pustejovsky JE, & Tipton E (2018). Small-sample methods for cluster-robust variance estimation and hypothesis testing in fixed effects models. Journal of Business & Economic Statistics, 36(4), 672–683.

[R134] Qi B, Fiori LM, Turecki G, & Trakadis YJ (2020). Machine learning analysis of blood microRNA data in major depression: A case-control study for biomarker discovery. International Journal of Neuropsychopharmacology, 23(8), 505–510. 32365192 10.1093/ijnp/pyaa029 PMC7689198

[R135] Ramasubbu R, Brown EC, Mouches P, Moore JA, Clark DL, Molnar CP, … Forkert ND (2024). Multimodal imaging measures in the prediction of clinical response to deep brain stimulation for refractory depression: A machine learning approach. The World Journal of Biological Psychiatry, 25(3), 175–187. 38185882 10.1080/15622975.2023.2300795

[R136] Rangaprakash D, Tadayonnejad R, Deshpande G, O’Neill J, & Feusner JD (2021). FMRI hemodynamic response function (HRF) as a novel marker of brain function: Applications for understanding obsessive-compulsive disorder pathology and treatment response. Brain Imaging and Behavior, 15, 1622–1640. 32761566 10.1007/s11682-020-00358-8 PMC7865013

[R137] Ravan M, Noroozi A, Gediya H, Basco KJ, & Hasey G (2024). Using deep learning and pretreatment EEG to predict response to sertraline, bupropion, and placebo. Clinical Neurophysiology, 167, 198–208. 39332081 10.1016/j.clinph.2024.09.002

[R138] Redlich R, Opel N, Grotegerd D, Dohm K, Zaremba D, Bürger C, … Dannlowski U (2016). Prediction of individual response to electroconvulsive therapy via machine learning on structural magnetic resonance imaging data. JAMA. Psychiatry, 73(6), 557 564. 27145449 10.1001/jamapsychiatry.2016.0316

[R139] Reggente N, Moody TD, Morfini F, Sheen C, Rissman J, O’Neill J, & Feusner JD (2018). Multivariate resting-state functional connectivity predicts response to cognitive behavioral therapy in obsessive–compulsive disorder. Proceedings of the National Academy of Sciences, 115(9), 2222–2227. 10.1073/pnas.1716686115 PMC5834692 29440404

[R140] Reitsma J, Glas A, Rutjes A, Scholten R, Bossuyt P, & Zwinderman A (2005). Bivariate analysis of sensitivity and specificity produces informative summary measures in diagnostic reviews. Journal of Clinical Epidemiology, 58, 982–990. 16168343 10.1016/j.jclinepi.2005.02.022

[R141] Rosellini AJ, Andrea AM, Galiano CS, Hwang I, Brown TA, Luedtke A, & Kessler RC (2023). Developing transdiagnostic internalizing disorder prognostic indices for outpatient cognitive behavioral therapy. Behavior Therapy, 54(3), 461–475. 37088504 10.1016/j.beth.2022.11.004 PMC10126479

[R142] Rosenthal R. (1991). Meta-analytic procedures for social research (Revised ed.). Newbury Park, CA: SAGE.

[R143] Rost N, Brückl TM, Koutsouleris N, Binder EB, & Müller-Myhsok B (2022). Creating sparser prediction models of treatment outcome in depression: A proof-of-concept study using simultaneous feature selection and hyperparameter tuning. BMC Medical Informatics and Decision Making, 22(1), 181. 35836174 10.1186/s12911-022-01926-2 PMC9284749

[R144] Rost N, Dwyer DB, Gaffron S, Rechberger S, Maier D, Binder EB, & Brückl TM (2023). Multimodal predictions of treatment outcome in major depression: A comparison of data-driven predictors with importance ratings by clinicians. Journal of Affective Disorders, 327, 330–339. 36750160 10.1016/j.jad.2023.02.007

[R145] Sajjadian M, Lam RW, Milev R, Rotzinger S, Frey BN, Soares CN, … Uher R (2021). Machine learning in the prediction of depression treatment outcomes: A systematic review and meta-analysis. Psychological Medicine, 51(16), 2742–2751. 35575607 10.1017/S0033291721003871

[R146] Sajjadian M, Uher R, Ho K, Hassel S, Milev R, Frey BN, … Kennedy SH (2023). Prediction of depression treatment outcome from multimodal data: A CAN-BIND-1 report. Psychological Medicine, 53(12), 5374–5384. 36004538 10.1017/S0033291722002124 PMC10482706

[R147] Salem H, Huynh T, Topolski N, Mwangi B, Trivedi MH, Soares JC, … Selvaraj S (2023). Temporal multi-step predictive modeling of remission in major depressive disorder using early stage treatment data; STAR* D based machine learning approach. Journal of Affective Disorders, 324, 286–293. 36584711 10.1016/j.jad.2022.12.076 PMC9863277

[R148] Semple DM, Suveges S, & Steele JD (2024). Electroconvulsive therapy response and remission in moderate to severe depressive illness: A decade of national Scottish data. The British Journal of Psychiatry, 225(6), 547–555. 39291460 10.1192/bjp.2024.126

[R149] Shahabi MS, Shalbaf A, Rostami R, & Kazemi R (2023). A convolutional recurrent neural network with attention for response prediction to repetitive transcranial magnetic stimulation in major depressive disorder. Scientific Reports, 13(1), 10147. 37349335 10.1038/s41598-023-35545-2 PMC10287753

[R150] Shumake J, Mallard TT, McGeary JE, & Beevers CG (2021). Inclusion of genetic variants in an ensemble of gradient boosting decision trees does not improve the prediction of citalopram treatment response. Scientific Reports, 11(1), 3780. 33580158 10.1038/s41598-021-83338-2 PMC7881144

[R151] Solomonov N, Lee J, Banerjee S, Flückiger C, Kanellopoulos D, Gunning FM, … Alexopoulos GS (2021). Modifiable predictors of nonresponse to psychotherapies for late-life depression with executive dysfunction: A machine learning approach. Molecular Psychiatry, 26(9), 5190–5198. 32651477 10.1038/s41380-020-0836-z PMC8120667

[R152] Squires M, Tao X, Elangovan S, Gururajan R, Zhou X, Li Y, & Acharya UR (2023). Identifying predictive biomarkers for repetitive transcranial magnetic stimulation response in depression patients with explainability. Computer Methods and Programs in Biomedicine, 242, Article 107771. 37717523 10.1016/j.cmpb.2023.107771

[R153] Sun J, Sun K, Chen L, Li X, Xu K, Guo C, … Fang J (2024). A predictive study of the efficacy of transcutaneous auricular vagus nerve stimulation in the treatment of major depressive disorder: An fMRI-based machine learning analysis. Asian Journal of Psychiatry, 104079. 38838458 10.1016/j.ajp.2024.104079

[R154] Sundermann B, Bode J, Lueken U, Westphal D, Gerlach AL, Straube B, … Pfleiderer B (2017). Support vector machine analysis of functional magnetic resonance imaging of interoception does not reliably predict individual outcomes of cognitive behavioral therapy in panic disorder with agoraphobia. Frontiers in Psychiatry, 8, 99. 28649205 10.3389/fpsyt.2017.00099 PMC5465291

[R155] Takamiya A, Liang KC, Nishikata S, Tarumi R, Sawada K, Kurokawa S, … Kishimoto T (2020). Predicting individual remission after electroconvulsive therapy based on structural magnetic resonance imaging: A machine learning approach. The Journal of ECT, 36(3), 205–210. 32118692 10.1097/YCT.0000000000000669

[R156] Taliaz D, Spinrad A, Barzilay R, Barnett-Itzhaki Z, Averbuch D, Teltsh O, … Lerer B (2021). Optimizing prediction of response to antidepressant medications using machine learning and integrated genetic, clinical, and demographic data. Translational Psychiatry, 11(1), 381. 34238923 10.1038/s41398-021-01488-3 PMC8266902

[R157] Tian S, Sun Y, Shao J, Zhang S, Mo Z, Liu X, … Lu Q (2020). Predicting escitalopram monotherapy response in depression: The role of anterior cingulate cortex. Human Brain Mapping, 41(5), 1249–1260. 31758634 10.1002/hbm.24872 PMC7268019

[R158] Tsai HJ, Yang WC, Tsai SJ, Lin CH, & Yang AC (2023). Right-side frontal-central cortical hyperactivation before the treatment predicts outcomes of antidepressant and electroconvulsive therapy responsivity in major depressive disorder. Journal of Psychiatric Research, 161, 377–385. 37012197 10.1016/j.jpsychires.2023.03.023

[R159] Tsai PL, Chang HH, & Chen PS (2022). Predicting the treatment outcomes of antidepressants using a deep neural network of deep learning in drug-naïve major depressive patients. Journal of Personalized Medicine, 12(5), 693. 35629117 10.3390/jpm12050693 PMC9146151

[R160] Tymofiyeva O, Yuan JP, Huang CY, Connolly CG, Blom EH, Xu D, & Yang TT (2019). Application of machine learning to structural connectome to predict symptom reduction in depressed adolescents with cognitive behavioral therapy (CBT). NeuroImage: Clinical, 23, Article 101914. 31491813 10.1016/j.nicl.2019.101914 PMC6627980

[R161] Vetter JS, Schultebraucks K, Galatzer-Levy I, Boeker H, Brühl A, Seifritz E, & Kleim B (2022). Predicting non-response to multimodal day clinic treatment in severely impaired depressed patients: A machine learning approach. Scientific Reports, 12(1), 5455. 35361809 10.1038/s41598-022-09226-5 PMC8971434

[R162] Viechtbauer W. (2010). Conducting Meta-analyses in R with the metafor package. Journal of Statistical Software, 36(3), 1–48.

[R163] Viechtbauer W. (2024). A fail-safe N computation based on the random-effects model. Amsterdam, The Netherlands: Annual Meeting of the Society for Research Synthesis Methodology.

[R164] Wallert J, Boberg J, Kaldo V, Mataix-Cols D, Flygare O, Crowley JJ, … Rück C (2022). Predicting remission after internet-delivered psychotherapy in patients with depression using machine learning and multi-modal data. Translational Psychiatry, 12(1), 357. 36050305 10.1038/s41398-022-02133-3 PMC9437007

[R165] Wang F, You Z, Zhang T, Xu K, Wang L, He J, & Tang J (2025). Predicting the treatment response of patients with major depressive disorder to selective serotonin reuptake inhibitors using machine learning techniques and EEG functional connectivity features. Depression and Anxiety, 2025(1), 9340993.

[R166] Wang J, Ouyang H, Jiao R, Cheng S, Zhang H, Shang Z, … Liu W (2024a). The application of machine learning techniques in posttraumatic stress disorder: A systematic review and meta-analysis. npj Digital Medicine, 7(1), 121. 38724610 10.1038/s41746-024-01117-5 PMC11082170

[R167] Wang J, Wu DD, DeLorenzo C, & Yang J (2024b). Examining factors related to low performance of predicting remission in participants with major depressive disorder using neuroimaging data and other clinical features. PLoS One, 19(3), Article e0299625. 38547128 10.1371/journal.pone.0299625 PMC10977765

[R168] Wang T, Gao C, Li J, Li L, Yue Y, Liu X, … Yuan Y (2024c). Prediction of early antidepressant efficacy in patients with major depressive disorder based on multidimensional features of rs-fMRI and P11 gene DNA methylation: Prédiction de l’efficacité précoce d’un antidépresseur chez des patients souffrant du trouble dépressif majeur d’après les caractéristiques multidimensionnelles de la méthylation de l’ADN du gène P11 et de la IRMf-rs. The Canadian Journal of Psychiatry, 69(4), 264–274. 37920958 10.1177/07067437231210787 PMC10924577

[R169] Wang Y, Zhou J, Ye J, Sun Z, He Y, Zhao Y, … Yang J (2023). Multi-omics reveal microbial determinants impacting the treatment outcome of antidepressants in major depressive disorder. Microbiome, 11(1), 195. 37641148 10.1186/s40168-023-01635-6 PMC10464022

[R170] Winter NR, Blanke J, Leenings R, Ernsting J, Fisch L, Sarink K, … Hahn T (2024). A systematic evaluation of machine learning–based biomarkers for major depressive disorder. JAMA Psychiatry, 81(4), 386–395. 38198165 10.1001/jamapsychiatry.2023.5083 PMC10782379

[R171] Xiao W, Moncy JC, Ghazi-Noori AR, Woodham RD, Rezaei H, Bramon E, … Fu CH (2025). Enhanced network synchronization connectivity following transcranial direct current stimulation (tDCS) in bipolar depression: Effects on EEG oscillations and deep learning-based predictors of clinical remission. Journal of Affective Disorders, 369, 576–587. 39293596 10.1016/j.jad.2024.09.054

[R172] Xu Z, Vekaria V, Wang F, Cukor J, Su C, Adekkanattu P, … Pathak J (2023). Using machine learning to predict antidepressant treatment outcome from electronic health records. Psychiatric Research and Clinical Practice, 5(4), 118–125. 38077277 10.1176/appi.prcp.20220015 PMC10698704

[R173] Yarkoni T, & Westfall J (2017). Choosing prediction over explanation in psychology: Lessons from machine learning. Perspectives on Psychological Science, 12(6), 1100–1122. 28841086 10.1177/1745691617693393 PMC6603289

[R174] Yun JY, Jang JH, Kim SN, Jung WH, & Kwon JS (2015). Neural correlates of response to pharmacotherapy in obsessive-compulsive disorder: Individualized cortical morphology-based structural covariance. Progress in NeuroPsychopharmacology and Biological Psychiatry, 63, 126–133. 10.1016/j.pnpbp.2015.06.009 26116795

[R175] Zandvakili A, Philip NS, Jones SR, Tyrka AR, Greenberg BD, & Carpenter LL (2019). Use of machine learning in predicting clinical response to transcranial magnetic stimulation in comorbid posttraumatic stress disorder and major depression: A resting state electroencephalography study. Journal of Affective Disorders, 252, 47–54. 30978624 10.1016/j.jad.2019.03.077 PMC6520189

[R176] Zhang H, Li X, Pang J, Zhao X, Cao S, Wang X, … Li H (2020). Predicting SSRI resistance: Clinical features and tagSNPs prediction models based on support vector machine. Frontiers in Psychiatry, 11, 493. 32581871 10.3389/fpsyt.2020.00493 PMC7283444

[R177] Zhao Z, Ran X, Niu Y, Qiu M, Lv S, Zhu M, … Yu Y (2025). Predicting treatment response of rTMS in major depressive disorder using a explainable machine learning model based on EEG and clinical features. Biological Psychiatry: Cognitive Neuroscience and Neuroimaging. 10.1016/j.bpsc.2025.02.002 39978464

[R178] Zhao Z, Ran X, Wang J, Lv S, Qiu M, Niu Y, … Yu Y (2024). Common and differential EEG microstate of major depressive disorder patients with and without response to rTMS treatment. Journal of Affective Disorders, 367, 777–787. 39265862 10.1016/j.jad.2024.09.040

[R179] Zhdanov A, Atluri S, Wong W, Vaghei Y, Daskalakis ZJ, Blumberger DM, … Farzan F (2020). Use of machine learning for predicting escitalopram treatment outcome from electroencephalography recordings in adult patients with depression. JAMA Network Open, 3(1), e1918377. 31899530 10.1001/jamanetworkopen.2019.18377 PMC6991244

[R180] Zhou S, Ma Q, Lou Y, Lv X, Tian H, Wei J, … Yu X (2021). Machine learning to predict clinical remission in depressed patients after acute phase selective serotonin reuptake inhibitor treatment. Journal of Affective Disorders, 287, 372–379. 33836365 10.1016/j.jad.2021.03.079

[R181] Zhukovsky P, Trivedi MH, Weissman M, Parsey R, Kennedy S, & Pizzagalli DA (2025). Generalizability of treatment outcome prediction across antidepressant treatment trials in depression. JAMA Network Open, 8(3), e251310. 40111362 10.1001/jamanetworkopen.2025.1310 PMC11926635

[R182] Zhutovsky P, Zantvoord JB, Ensink JB, op den Kelder R, Lindauer RJ, & van Wingen GA (2021). Individual prediction of trauma-focused psychotherapy response in youth with posttraumatic stress disorder using resting-state functional connectivity. NeuroImage: Clinical, 32, Article 102898. 34911201 10.1016/j.nicl.2021.102898 PMC8645516

[R183] Ziobrowski HN, Cui R, Ross EL, Liu H, Puac-Polanco V, Turner B, … Kessler RC (2023). Development of a model to predict psychotherapy response for depression among veterans. Psychological Medicine, 53(8), 3591–3600. 35144713 10.1017/S0033291722000228 PMC9365879

