# Supplementary material for: Machine learning in the prediction of treatment response for emotional disorders: A systematic review and meta-analysis
Source: Clin Psychol Rev. Author manuscript; Available in PMC 2026 Feb 18. (PMC12915758; doi:10.1016/j.cpr.2025.102593)
Supplement: Supplement Methods and Results [file NIHMS2139823-supplement-Supplement_Methods_and_Results.docx]

**Database Search Terms**

A comprehensive literature search was conducted across PubMed and PsycINFO databases from January 1^st^, 2010, to July 23^rd^, 2024. The comprehensive list of search terms including logical operators were as follows:

(machine learning OR machine learn* OR deep learning OR deep learn*)

AND

(treatment OR psychotherapy OR cognitive behavior therapy OR CBT OR cognitive therapy OR therapy OR mindfulness OR dialectical behavior therapy OR DBT OR exposure therapy OR medication OR pharmacotherapy OR pharmaco* OR acceptance and commitment therapy OR biofeedback)

AND

((Anxiety OR anxiety disorders OR anxiety disorder OR anxious OR panic OR panic disorder OR agoraphobia OR social phobia OR social anxiety OR social anxiety disorder OR sad OR generalized anxiety OR gad OR general anxiety disorder OR obsessive compulsive OR obsessive-compulsive OR ocd OR obsessive compulsive disorder OR obsessive compulsive disorder OR specific phobia OR simple phobia OR phob* OR post-traumatic stress OR posttraumatic stress OR ptsd OR acute stress OR posttraumatic stress OR post-traumatic stress disorder OR posttraumatic stress disorder OR post traumatic stress disorder OR asd OR body dysmorphic disorder OR BDD OR eating disorder* OR anorexia OR bulimia) OR (depression OR depressive OR depress* OR major depressive disorder OR MDD))

**Supplementary Methods and Results**

*Interaction between sample size and algorithm type on prediction accuracy*

**Methods**

A further moderator analysis was conducted to examine whether there was an interaction effect between study sample size (N) and class of machine learning algorithm. The same meta-regression procedure was used as described in the body of the paper with exponentiated coefficients being presented. Furthermore, the regression algorithm was used as the reference category, and random slopes were used in addition to random intercepts.

**Results and Discussion**

Overall, the results suggested there may be a significant interaction effect between sample size and the comparison between neural networks and regression (*b* = 1.0008, p < 0.001). That is, neural network algorithms are associated with greater prediction accuracy under conditions of increased sample sizes. This is perhaps not entirely surprising, given that neural networks and deep learning algorithms typically require large sample sizes (Kuhn & Johnson, 2013). Of note, there was another interaction effect between sample size and the effect of a nondescript class of other algorithms (e.g., Generalized Naive Bayes Classifier, etc.) that do not concisely fit with other overarching algorithm types. Specifically, this class of algorithms generally (*b* = 0.9978, p < 0.001) performs less well with increases in sample size.

Supplementary Table 1: Results of interaction between sample size and algorithm type on prediction accuracy

| Term | *b* | *SE* | *t* | *p* | 95% CI |
| --- | --- | --- | --- | --- | --- |
| N | 0.9998 | 0.00 | -0.81 | .415 | [0.9993, 1.0002] |
| Algorithm (GB) | 0.74 | 0.55 | -0.56 | .577 | [0.25, 2.15] |
| Algorithm (Neural Net) | 0.50 | 0.49 | -1.39 | .163 | [0.19, 1.32] |
| Algorithm (Other) | 2.01 | 0.85 | 0.82 | .411 | [0.38, 10.61] |
| Algorithm (SVM) | 1.12 | 0.39 | 0.30 | .766 | [0.53, 2.39] |
| Algorithm (Tree) | 1.07 | 0.49 | 0.14 | .887 | [0.41, 2.78] |
| N x Algorithm (GB) | 1.0002 | 0.00 | 0.32 | .750 | [0.99, 1.0001] |
| N x Algorithm (Neural Net) | 1.0008 | 0.00 | 3.48 | < .001*** | [1.0003, 1.001] |
| N x Algorithm (Other) | 0.9978 | 0.00 | -2.47 | <.05* | [0.9961, 0.9995] |
| N x Algorithm (SVM) | 0.9996 | 0.00 | -0.69 | .488 | [0.9984, 1.0008] |
| N x Algorithm (Tree) | 0.9998 | 0.00 | -1.03 | .302 | [0.9994, 1.0002] |

*Note*: Definitions of abbreviations are as follows: *b* = Beta Value; *SE* = Standard Error; CI = Confidence Interval; GB = Gradient Boosting; SVM = Support Vector Machine. All coefficients are exponentiated values to represent odds ratios.

Supplementary Figure 1: Forest Plot of Nested Effects

Insert Supplementary Figure here

*Notes*: These effects report each effect nested within each study, as determined by the multilevel random-effects model meta-analysis. Please note there might be slight discrepancies in the accuracy values in the Forest Plot and Table 1 due to rounding error. The meta-analyzed results presented in the current figure represent the accuracies calculated from inverse logit back-transformation.
