# Supplementary material for: Machine learning in the prediction of treatment response for emotional disorders: A systematic review and meta-analysis
Source: Clin Psychol Rev. Author manuscript; Available in PMC 2026 Feb 18. (PMC12915758; doi:10.1016/j.cpr.2025.102593)
Supplement: Supplement Table [file NIHMS2139823-supplement-Supplement_Table.docx]

| Term | *b* | *SE* | *t* | *p* | 95% CI |
| --- | --- | --- | --- | --- | --- |
| N | 0.9998 | 0.00 | -0.81 | .415 | [0.9993, 1.0002] |
| Algorithm (GB) | 0.74 | 0.55 | -0.56 | .577 | [0.25, 2.15] |
| Algorithm (Neural Net) | 0.50 | 0.49 | -1.39 | .163 | [0.19, 1.32] |
| Algorithm (Other) | 2.01 | 0.85 | 0.82 | .411 | [0.38, 10.61] |
| Algorithm (SVM) | 1.12 | 0.39 | 0.30 | .766 | [0.53, 2.39] |
| Algorithm (Tree) | 1.07 | 0.49 | 0.14 | .887 | [0.41, 2.78] |
| N x Algorithm (GB) | 1.0002 | 0.00 | 0.32 | .750 | [0.99, 1.0001] |
| N x Algorithm (Neural Net) | 1.0008 | 0.00 | 3.48 | < .001*** | [1.0003, 1.001] |
| N x Algorithm (Other) | 0.9978 | 0.00 | -2.47 | <.05* | [0.9961, 0.9995] |
| N x Algorithm (SVM) | 0.9996 | 0.00 | -0.69 | .488 | [0.9984, 1.0008] |
| N x Algorithm (Tree) | 0.9998 | 0.00 | -1.03 | .302 | [0.9994, 1.0002] |
