# Supplementary figures and images for: Machine learning in the prediction of treatment response for emotional disorders: A systematic review and meta-analysis
Source: Clin Psychol Rev. Author manuscript; Available in PMC 2026 Feb 18. (PMC12915758; doi:10.1016/j.cpr.2025.102593)

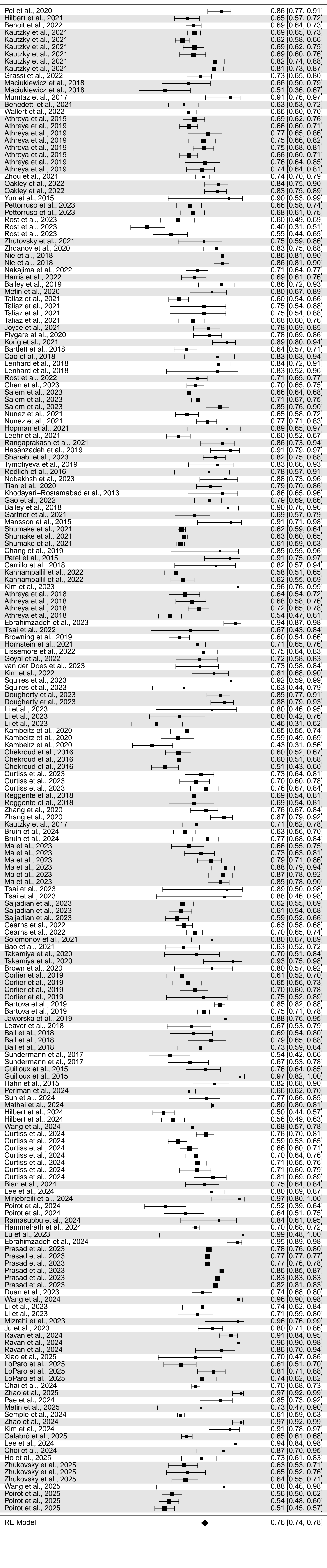

Supplement: Supplement Fig 1 [file NIHMS2139823-supplement-Supplement_Fig_1.pdf]
